# The dynamic trophic architecture of open-ocean protist communities revealed through machine-guided metatranscriptomics

**DOI:** 10.1073/pnas.2100916119

**Published:** 2022-02-10

**Authors:** Bennett S. Lambert, Ryan D. Groussman, Megan J. Schatz, Sacha N. Coesel, Bryndan P. Durham, Andrew J. Alverson, Angelicque E. White, E. Virginia Armbrust

**Affiliations:** ^a^School of Oceanography, University of Washington, Seattle, WA 98195;; ^b^Department of Biology, University of Florida, Gainesville, FL 32611;; ^c^Genetics Institute, University of Florida, Gainesville, FL 32611;; ^d^Department of Biological Sciences, University of Arkansas, Fayetteville, AR 72701;; ^e^Department of Oceanography, University of Hawai’i at Manoa, Honolulu, HI 96822

**Keywords:** machine learning, mixotrophy, metatranscriptomics, microbial ecology, trophic mode

## Abstract

Intricate networks of single-celled eukaryotes (protists) dominate carbon flow in the ocean. Their growth, demise, and interactions with other microorganisms drive the fluxes of biogeochemical elements through marine ecosystems. Mixotrophic protists are capable of both photosynthesis and ingestion of prey and are dominant components of open-ocean planktonic communities. Yet the role of mixotrophs in elemental cycling is obscured by their capacity to act as primary producers or heterotrophic consumers depending on factors that remain largely uncharacterized. Here, we develop and apply a machine learning model that predicts the in situ trophic mode of aquatic protists based on their patterns of gene expression. This approach leverages a public collection of protist transcriptomes as a training set to identify a subset of gene families whose transcriptional profiles predict trophic mode. We applied our model to nearly 100 metatranscriptomes obtained during two oceanographic cruises in the North Pacific and found community-level and population-specific evidence that abundant open-ocean mixotrophic populations shift their predominant mode of nutrient and carbon acquisition in response to natural gradients in nutrient supply and sea surface temperature. Metatranscriptomic data from ship-board incubation experiments revealed that abundant mixotrophic prymnesiophytes from the oligotrophic North Pacific subtropical gyre rapidly remodeled their transcriptome to enhance photosynthesis when supplied with limiting nutrients. Coupling this approach with experiments designed to reveal the mechanisms driving mixotroph physiology provides an avenue toward understanding the ecology of mixotrophy in the natural environment.

## Significance

Mixotrophy is a ubiquitous nutritional strategy in marine ecosystems. Although our understanding of the distribution and abundance of mixotrophic plankton has improved significantly, the functional roles of mixotrophs are difficult to pinpoint, as mixotroph nutritional strategies are flexible and form a continuum between heterotrophy and phototrophy. We developed a machine learning–based method to assess the nutritional strategies of in situ planktonic populations based on metatranscriptomic profiles. We demonstrate that mixotrophic populations play varying functional roles along physicochemical gradients in the North Pacific Ocean, revealing a degree of physiological plasticity unique to aquatic mixotrophs. Our results highlight mechanisms that may dictate the flow of biogeochemical elements and ecology of the North Pacific Ocean, one of Earth's largest biogeographical provinces.

Single-celled eukaryotes (protists) form the base of marine food webs and their growth, demise, and interactions with other microorganisms drive the biogeochemistry of the Earth’s oceans (1, 2). Marine protists are genetically and functionally diverse, with representatives from all major lineages of the eukaryotic tree of life (3). Some protists are predatory heterotrophs that obtain organic carbon and nutrients through the ingestion of prey. They are commonly motile, consume smaller prey via phagocytic engulfment (4, 5), and may consume larger prey through veil feeding (6). Once engulfed, prey-derived nutrients are absorbed in acidic vacuoles (4, 6, 7). Other protists are strictly photosynthetic, with the ability to biosynthesize required organic compounds from inorganic carbon and nutrients with sunlight as an energy source. Photosynthesis requires a cellular organization and composition distinct from that of heterotrophs. A growing list of marine protists are described as mixotrophic and are capable of both photosynthesis and phagocytic feeding (8, 9), a trait combination rarely observed in terrestrial ecosystems (10). Marine mixotrophs are broadly distributed across the major eukaryotic lineages (7–9, 11–15), including many species that form harmful algal blooms (7).

Despite the pervasiveness of mixotrophy in marine systems, generalizations concerning the ecology of these organisms remain elusive, given their diversity and the relative paucity of available physiological and biogeographical data. An early “eat-your-competitor” hypothesis (16) suggested that by consuming bacteria, mixotrophs reduce competition for dissolved nutrients. Subsequent experimental studies found that mixotrophic species can outcompete heterotrophic specialists by grazing prey down to abundances below the critical threshold required for the survival of the specialist (17, 18). The mixotrophic consumption of bacteria may also benefit photosynthetic organisms (19) by reducing competition for dissolved nutrients. Nutrient-limiting conditions and sufficient light may provide mixotrophs with a further competitive advantage over trophic specialists (17) through their ability to combine grazing and photosynthesis, although subsequent studies documented species-specific differences in mixotroph grazing behavior (20) and differential grazing responses to light and nutrient limitation (21, 22). In the natural environment, mixotroph abundance is estimated via feeding experiments in which sampled communities are incubated in bottles with fluorescently labeled bacteria added as a prey source. Through the microscopic enumeration of cells that exhibit both chlorophyll autofluorescence and fluorescence due to ingested prey (9, 23), mixotrophic plankton have been detected in nutrient-rich coastal waters (12, 14, 24), in coastal and open-ocean oligotrophic systems where dissolved nutrient availability is low (25, 26), and in light-limited polar waters where mixotrophy may serve as a key survival tactic for overwintering (22, 27). Within these environments, mixotrophs account for an estimated 40 to >80% of the nanoplankton (2 to 20 µm) and 35 to 95% of detected bacterivory (25, 26).

The inclusion of mixotrophic organisms that simultaneously photosynthesize and phagocytose into global ecosystem models dramatically alters modeled food web dynamics and carbon cycling with an increase in the average cell size in planktonic communities, increased efficiency of carbon transfer across trophic levels, and enhanced carbon export from surface waters (28, 29). Modeled patterns of nitrogen acquisition suggest that the phagocytic acquisition of nitrogen in oligotrophic subtropical gyres shifts to diffusive uptake in more nutrient-rich waters. In a direct link between field observations and model simulations, Edwards (24) compiled data from over 100 environmental feeding assays conducted in a variety of environments and interpreted the data within a resource allocation model framework that balanced carbon and nutrient requirements under different trophic modes. He proposed that mixotrophs outcompete obligate heterotrophs when the ratio of prey to nutrient availability is low, as mixotrophs can relieve potential carbon limitation via photosynthesis. Edwards argued further that, under conditions of nutrient limitation, mixotrophs outcompete autotrophs because of their ability to consume prey. Edwards thus predicted that mixotrophs will increase in relative abundance at low latitudes and in nutrient-rich coastal environments while recognizing that strategies for any individual organism may vary across biogeochemical gradients. Together, these models show the potential importance of mixotrophy in marine ecosystems, although the lack of high-resolution observations from diverse regions means the parametrizations of mixotrophy remain simplistic.

The available data emphasize the diversity, trophic flexibility, and importance of mixotrophs to marine ecosystem function. However, the diversity of mixotrophic organisms and their species-specific trophic responses to environmental conditions make it difficult to characterize their behavior in situ. We approached this problem with the premise that different trophic modes would produce distinctive gene expression profiles. Traditionally, differentially transcribed genes under a given set of physiological conditions are identified via a comparative approach, a process that tends to select for the most highly expressed genes in an organism. Machine learning techniques are more suited to the identification of trends within multidimensional datasets, including transcriptional patterns across multiple species and conditions. We hypothesized that trophic mode-specific patterns of gene expression could be identified through the development of a machine learning model, despite limited knowledge of the underlying molecular basis for these differences. We leveraged a large public database of transcriptomes from heterotrophic, mixotrophic, and photosynthetic aquatic protist species (30) to develop, train, and test a trophic mode prediction model using machine learning approaches. The application of this model to metatranscriptomes acquired along natural biogeochemical gradients in the North Pacific Ocean and analysis of samples from at-sea nutrient addition experiments predict that mixotrophic protists shift their predominant mode of nutrient and carbon acquisition in response to natural gradients in nutrient supply and sea surface temperature.

## Results

### A Machine Learning Approach to Predict Protistan Trophic Mode.

To determine whether transcriptional profiles could be used to distinguish between different trophic modes, we developed a pipeline (*SI Appendix*, Fig. S1) that leverages transcriptomes derived from marine protists cultured in conditions that select for a known trophic mode to generate a training dataset (*SI Appendix*, Fig. S1, *Dataset Preparation*). We distilled this training dataset into the gene families that drive trophic mode prediction accuracy (*SI Appendix*, Fig. S1, *Feature Selection*) and used these gene families to generate a predictive trophic mode model (*SI Appendix*, Fig. S1, *Model Training*). Before applying the predictive model to environmental data, model limits were determined using external validation transcriptome datasets, and the function and expression patterns of the selected gene families within the training set were assessed (*SI Appendix*, Fig. S1, *Model Evaluation*).

The transcriptome training dataset was derived from the 656 transcriptomes of marine protists publicly available through the Marine Microbial Eukaryote Transcriptome Sequencing Project (MMETSP) (30, 31). Trophic mode labels could be assigned to ∼70% of the individual transcriptomes based on provided growth conditions and available literature (unlabeled transcriptomes either had insufficient metadata or conflicting literature-based classifications), allowing more than one trophic mode to be assigned to a species depending on growth conditions and thus partially decoupling trophic mode from phylogeny (Dataset S1 and File S1; https://github.com/armbrustlab/trophic-mode-ml/blob/main/FileS1.html). For example, an individual transcriptome was labeled as mixotrophic if a known mixotroph was grown in the light and in the presence of bacteria or as phototrophic if the same species was grown in the light in the absence of bacteria. The resulting training set consisted of 446 labeled transcriptomes (275 derived from phototrophic growth conditions, 93 from mixotrophic, and 78 from heterotrophic conditions) and encompassed more than 9,000 gene families (Pfams) (32). Categorizing our training set in this manner allowed us to seek out representative features of trophic mode across diverse organisms.

**Fig. 1.**
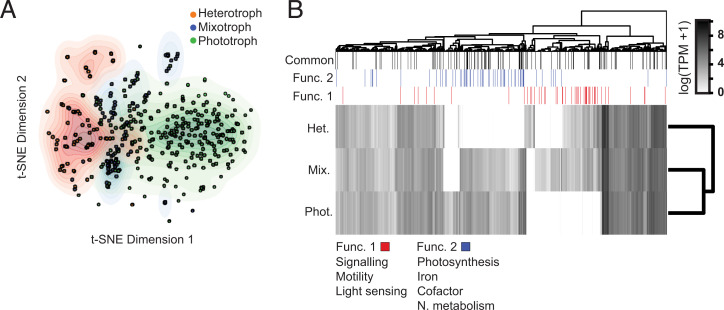
Feature selection helps distinguish trophic modes. (*A*) Latent representation of MMETSP phototrophic, heterotrophic, and mixotrophic transcriptomes based on common selected features (*f* = 120). Transcriptional profiles of selected gene families were scaled and transformed through t-SNE. Color contours indicate kernel density for each trophic mode. (*B*) Median transcript abundances of the combined set of selected genes (*f* = 1,046) in the MMETSP highlight clusters of gene families that broadly differentiate trophic modes. The gene family annotations indicate those enriched in heterotrophic/mixotrophic (Func. 1), phototrophic/mixotrophic (Func. 2), and those present in the common set of selected features. TPM: Transcripts per million.

Gene families that impacted trophic mode classification were identified within the training dataset via an in-house implementation of the train-test mean decrease in accuracy (MDA) algorithm (33) using two common tree-based classification algorithms: Random Forest (34) and XGBoost (35) (*SI Appendix*, Fig. S1, *Feature Selection*). MDA functions by iteratively training the model with a single gene family’s transcription values randomly permuted. If the shuffling of the gene family resulted in any decrease in model prediction accuracy, the gene family was retained for trophic mode classification as part of a reduced feature set. All other gene families were subsequently removed from the transcriptome training set. Accuracy was determined via cross-validation, a process by which the model is applied to data not used during training as a test of model skill. Our in-house implementation varies from that in ref. 33 in that cross-validation is used to compute accuracy and not a single test set. This aggressive pruning approach was used to avoid potential instabilities that can result from imbalances between the number of features (9,000 gene families) and the number of samples (446 transcriptomes) present in a training set (36). A total of 901 (Random Forest) or 265 (XGBoost) candidate gene families were retained and categorized either as a common set of gene families (*f* = 120; overlap between XGBoost and Random Forest sets) or a combined set of gene families (*f* = 1046; combination of XGBoost and Random Forest sets). Compared with a traditional differential expression analysis, very few of the selected gene families (5%) were among the most differentially transcribed gene families between trophic modes. This highlighted that our model effectively used gene families for classification that were difficult to extract from the data in a conventional manner. Regardless of which of the two reduced sets of gene families was used, heterotrophic and phototrophic transcriptomes were well separated in a t-distributed stochastic neighbor embedding (t-SNE) (37) latent space with mixotrophic transcriptomes partially overlapping both clusters (Fig. 1*A* and *SI Appendix*, Figs. S1, *Visualization*, and S2). This separation of trophic modes was most apparent when t-SNE was applied with the common set of gene families (*f* = 120; Fig. 1*A*) rather than all gene families (*f* = ∼9,000; *SI Appendix*, Fig. S2*D*), emphasizing the importance of identifying a reduced set of gene families. We chose to proceed with the tree-based models, as they have the additional attribute of robustness to potential multicollinearity among gene families (36) (*SI Appendix*, Fig. S3) and compared favorably to several simple classifiers (Dataset S2).

The combined set of 1,046 gene families encoded a variety of protein families, including those involved in photosynthesis, flagellar motility, and carbohydrate metabolism (Dataset S3; Function derived from Pfam summary). Those gene families with a nonzero median expression (*f* = 886/1,046) were hierarchically clustered across the MMETSP training set (Dataset S4) to determine whether they were differentially transcribed depending on trophic mode (Fig. 1*B*). Genes families enriched in heterotrophic transcriptomes encoded proteins involved in signaling and cell cycle processes, whereas gene families enriched in phototrophic transcriptomes encoded proteins involved in lipid metabolism. Interestingly, mixotrophs displayed a higher median transcription of a variety of genes that encode carbohydrate active enzymes (38), suggesting that mixotrophs process carbohydrates in a different manner than trophic specialists. Most clusters of enriched gene families were shared either between phototrophs and mixotrophs or between heterotrophs and mixotrophs. Clusters of gene families with similar relative transcript abundances within phototrophs and mixotrophs were associated with photosynthesis, cofactor synthesis, iron binding, and nitrogen metabolism, underscoring the metabolic cost of maintaining active photosystems (Fig. 1*B*, Func. 2). Clusters of gene families with similar relative transcript abundances in heterotrophs and mixotrophs encoded functions such as cell motility, light sensing, and signaling (Fig. 1*B*, Func. 1). This is consistent with the idea that mixotrophs and heterotrophs employ motility to increase encounters with prey, rely on chemotaxis and mechanosensing to recognize prey (39, 40), and are likely attuned to the light environment to maintain exposure to light and phototrophic prey. Additional gene families not clearly associated with a given trophic mode included regulatory proteins, ribosomal proteins, and proteins involved in protein degradation. Retraining models with Func. 1 and Func. 2 gene families resulted in models with a similar performance to those trained on the combined set of gene families (Datasets S2 and S5), highlighting potential redundancy of features in the combined set of gene families. The common set of 120 gene families clustered in similar patterns and encoded much of the functional diversity found in the combined set (Fig. 1*B*, Datasets S4 and S6, and *SI Appendix*, Fig. S4). The larger combined feature set was retained for further evaluation in order to retrieve as much information content as possible from environmental transcriptomes in which gene family sparsity is more pronounced than in laboratory cultures (*SI Appendix*, *Investigating factors that may impact prediction quality for environmental transcriptome bins*).

**Fig. 2.**
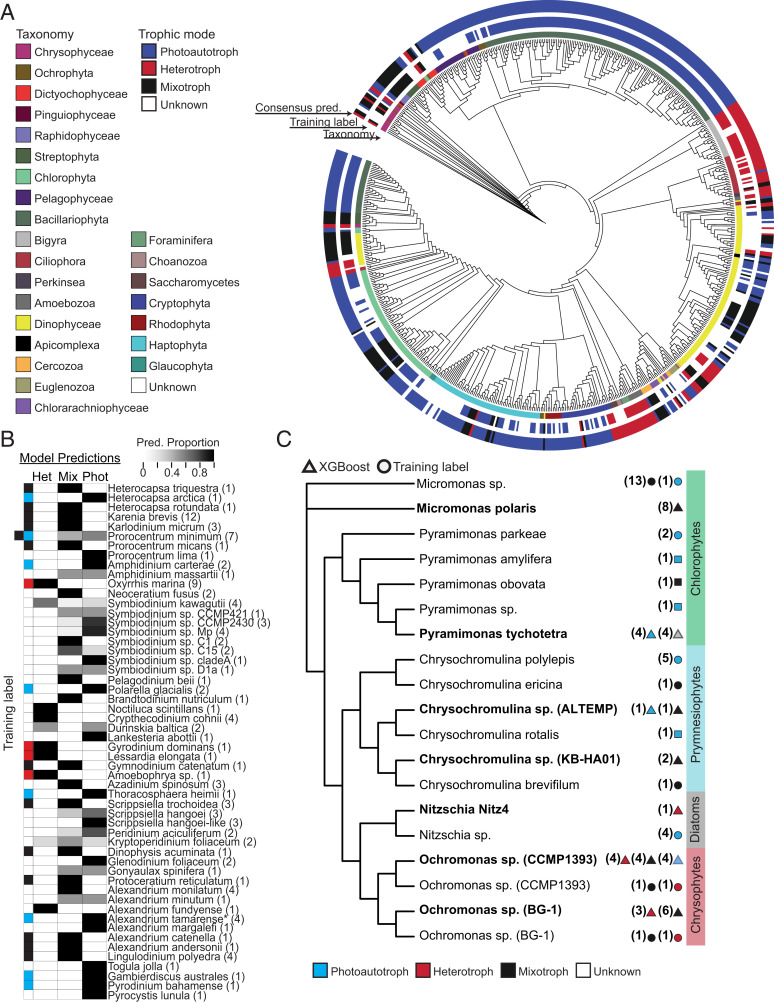
Trophic mode predictions for MMETSP transcriptomes and culture-derived validation transcriptomes. (*A*) Phylogenetic representation of taxa in the MMETSP database. Color strips surrounding the tree indicate (from inner to outer) taxonomic group membership, transcriptome trophic mode training label (if present), and consensus trophic mode prediction between Random Forest and XGBoost (if present) using the combined set of selected gene families. (*B*) Dinoflagellate taxa in the MMETSP database. From left to right: training labels when available (blue: phototrophy; red: heterotrophy; black: mixotrophy) and the proportion of trophic mode (het: heterotrophic, mix: mixotrophic, and phot: phototrophic) predictions derived from XGBoost model output using both selected feature sets; number of available MMETSP transcriptome replicates indicated in parentheses. *A. tamarense grown axenically and *P. minimum* grown under either phototrophic or mixotrophic culture conditions. (*C*) Phylogenetic representation of validation taxa (bold) with diverse trophic modes and closely related MMETSP representatives. *Nitzschia* sp. Nitz4 is a nonphotosynthetic diatom. *Ochromonas* sp. (strains BG-1 and CCMP1393), *Micromonas polaris* and *Pyramimonas tychotetra* have the capacity for mixotrophy. The values in parentheses indicate number of available MMETSP transcriptomes. Colored circles indicate trophic mode labels in the MMETSP training set. Colored squares indicate predictions for unlabeled MMETSP transcriptomes. Colored triangles indicate XGBoost predictions for unlabeled transcriptomes; majority vote shown for replicated transcriptomes. Translucent triangles indicate that the condition was present, but XGBoost made an incorrect prediction. See *SI Appendix*, Fig. S7 for a comprehensive overview of model performance on validation taxa.

We evaluated predictions derived from Random Forest and XGBoost models for all MMETSP transcriptomes, including the 210 transcriptomes that were not part of the original training set (Dataset S7 and *SI Appendix*, Fig S1, *Model Evaluation*) An 18S ribosomal DNA phylogenetic tree was constructed representing each MMETSP transcriptome, and the tree leaves were labeled by a predicted trophic mode based either on our literature review or an agreement between predictions derived from the two models (Fig. 2*A* and Dataset S1). Trophic mode predictions for unlabeled transcriptomes were in the greatest agreement between models for phyla dominated by either phototrophs or heterotrophs (Fig. 2*A*), whereas a small proportion (10%) of transcriptomes derived primarily from dinoflagellate and haptophyte species received inconsistent trophic mode predictions between models. To examine prediction consistency between replicate dinoflagellate transcriptomes, we aggregated model predictions to generate distributions of predictions for dinoflagellate transcriptomes present in the MMETSP (Fig. 2*B*). Upon closer inspection, 12 unlabeled dinoflagellate transcriptomes (25% of total) received either a combination of mixotrophic and heterotrophic classifications or mixotrophic and phototrophic classifications. *Kryptoperidinium foliaceum* and *Symbiodinium kawagutii* received model predictions split across all trophic modes, and *Durinskia baltica* received classifications split between heterotrophy and phototrophy. All other dinoflagellate transcriptomes were consistently classified (XGBoost: Fig. 2*B*; Random Forest: *SI Appendix*, Fig. S5) as either phototrophic (*n* = 16), heterotrophic (*n* = 9), or mixotrophic (*n* = 17). Finally, we tested MMETSP transcriptomes from known osmoheterotrophs, which gain carbon by taking up dissolved compounds rather that by consuming prey. All were predicted to be heterotrophs. This process identified a cluster of gene families related to chemical sensing, vacuoles, and motility that are transcribed by phagoheterotrophs and mixotrophs but not by osmoheterotrophs (*SI Appendix*, Fig. S6 and Dataset S8), suggesting that these genes may be associated with phagotrophy.

Finally, we challenged the models with additional transcriptomes that were not part of the MMETSP dataset (summary in Fig. 2*C*; complete results in *SI Appendix*, Fig. S7) (Fig. S1, *Model Evaluation*). Both models successfully classified a transcriptome from the nonphotosynthetic diatom *Nitzschia* sp. (Nitz4) (41) as heterotrophic despite the fact that all other diatoms within the training dataset were phototrophic. We evaluated available transcriptomes for three organisms (*Micromonas polaris*, *Pyramimonas tychotetra*, and two *Ochromonas* species) that are expected to shift trophic modes under different growth conditions (Dataset S9) (42). Both models accurately classified the organisms as mixotrophic under conditions in which cells were observed to ingest particles or as phototrophic under high nutrient conditions (Fig. 2*C*, colored triangles). However, the models did not consistently predict the expected trophic mode either when cells were grown in the absence of bacteria, which was expected to induce phototrophy, or under low nutrient conditions, which was expected to induce mixotrophy (Fig. 2*C*, translucent triangles). This inconsistency could reflect limited bacterial grazing documented in the low nutrient conditions (42), absence of representative mixotrophic *Pyramimonas* sp. transcriptomes in the training dataset, or the possibility that these organisms may not uniformly behave in culture as expected. Last, we generated and classified transcriptomes from two *Chrysochromulina* species isolated from near Hawaii in the North Pacific subtropical gyre. *Chrysochromulina* strain KB-HA01 was predicted to be mixotrophic, and AL-TEMP had predictions split between phototrophy and mixotrophy, consistent with observed mixotrophy in other *Chrysochromulina* sp. and our own observations (*SI Appendix*, Fig. S8).

Collectively, these tests identified several elements that impact model performance. First, XGBoost consistently outperformed Random Forest when applied to the validation transcriptomes (81 versus 74% accuracy) regardless of which reduced set of gene families was used (Cohen’s κ = 0.90 versus 0.93; Dataset S7). Second, both sets of selected gene families included those with either no known function or a predicted function with no obvious relation to trophic mode. Based on these outcomes, XGBoost and the combined set (1,046) of gene families were chosen for subsequent analyses. Third, prediction accuracy and consistency increased when predictions were made for organisms closely related to those present in the MMETSP training set. Fourth, model interpretability was improved through increased numbers of transcriptome replicates, often by yielding a clear majority vote prediction across replicates (Fig. 2*B*). Those experiments with few replicates were difficult to interpret. Replication is of even greater importance for field-based transcriptome data in which transcriptome completeness can vary between samples (*SI Appendix*), potentially introducing uncertainty into the model behavior. Lastly, in those instances in which replicate transcriptomes resulted in predictions that included both phototrophy and heterotrophy, we assumed that these transcriptional profiles were in conflict with the decision boundaries of our model and that the trophic mode could not be predicted accurately for these transcriptomes.

### Predicting the In Situ Trophic Mode of Marine Planktonic Populations.

Metatranscriptomes provide a snapshot of community function and metabolism and can be deconvolved into transcriptomes associated with representative taxonomic bins (*SI Appendix*, Fig. S9). A total of 26 species-level taxonomic bins were derived from 95 eukaryotic (polyA-selected) metatranscriptomes collected on two field campaigns in the North Pacific Ocean and were analyzed for in situ trophic status (*SI Appendix*, Fig. S1, *Model Deployment*). This analysis was restricted to taxonomic bins closely related to transcriptomes present in the original training set and with a minimum of four transcriptomes that met established completeness criteria (*SI Appendix*, *Investigating factors that may impact prediction quality for environmental transcriptome bins*) at any given sampling site.

Samples for 48 metatranscriptomes were collected during the Simons Collaboration on Ocean Processes and Ecology (SCOPE) Diel cruise (KM1513; HOE-Legacy 2, July/Aug 2015; *SI Appendix*, Fig. S10) near Station ALOHA (A Long-Term Oligotrophic Habitat Assessment) (158° W, 22.75° N) in the North Pacific subtropical gyre, a low latitude oligotrophic region where the availability of nitrogen limits productivity in surface waters and where ecosystem models (29) and observational studies (23, 25, 43) indicate the potential for abundant and active mixtotrophic organisms. Duplicate samples (0.2 to 100 µm) were collected from the same water mass (by following a Lagrangian drifter) at a depth of 15 m every 4 h over 4 d. A total of 18 species-level taxonomic bins (Dataset S10 and *SI Appendix*, Fig. S11) were retrieved (*SI Appendix*, *Methods*) that met our established completeness criteria (*SI Appendix*, *Investigating factors that may impact prediction quality for environmental transcriptome bins*), including the detection of transcripts associated with the selected feature set (1,023 of the 1,046 features were detected in the taxonomic bin transcriptomes). The trophic mode predictions were made for individual transcriptomes and summed for each taxonomic bin at a given location to generate trophic mode prediction proportions. Most (14/18) taxonomic bins were present throughout the day–night cycle of the 4-d sampling period and were each represented by at least 32 transcriptomes. A total of 16 taxonomic bins corresponded to the reference species with the potential to exhibit mixotrophy. Thus, the majority of these environmental species had the potential to exhibit diverse nutritional strategies.

**Fig. 3.**
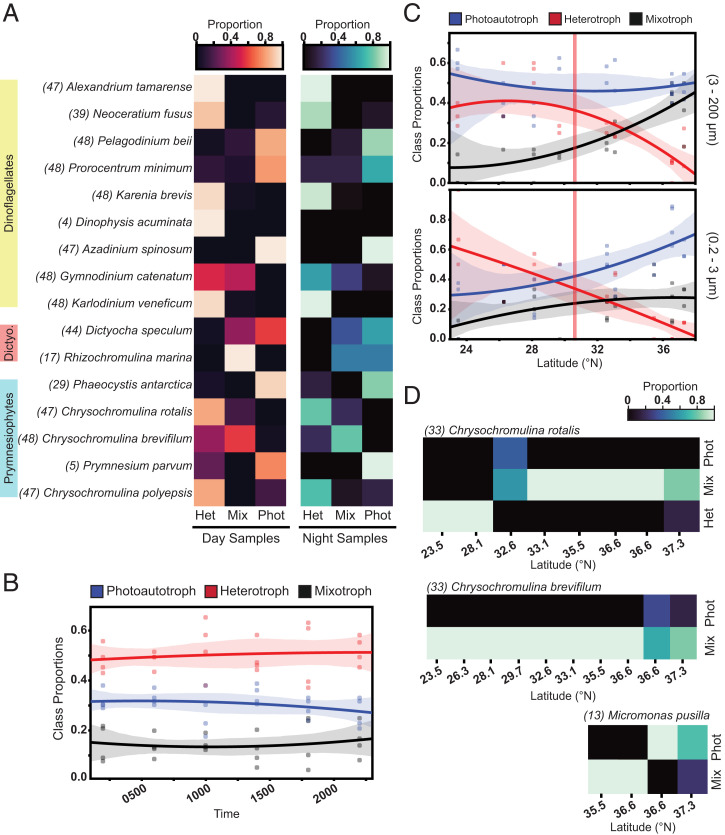
The distribution of model predictions across environmental taxonomic bins. (*A*) Heatmaps of the proportion of XGBoost-predicted trophic modes for each diel (KM1513, July/Aug. 2015, 22°N) transcriptome bin for samples taken during the day (*Left*) versus night (*Right*). Number of transcriptional profiles classified per taxonomic bin indicated in parentheses. (*B*) Proportion of trophic mode predictions (derived from *A*) over a diel cycle. Individual points represent samples obtained at the same time of day (*n* = 4). (*C*) Gradients 1 (KOK1606, April/May 2016) trophic mode prediction proportions summed by latitude and normalized by total number of predictions at each site. Solid lines: second order regressions; points: individual station values; shaded regions: 95% CIs; red vertical line: salinity front near 30° N. (*D*) Proportion of trophic mode predictions for three abundant protists across the G1 transect. Number of transcriptional profiles classified per taxonomic bin indicated in parentheses.

Nine environmental dinoflagellate bins were predicted to behave primarily as either heterotrophs (*n* = 6) or as phototrophs (*n* = 3) regardless of whether the transcriptomes were derived from day or night samples (Fig. 3*A* and Dataset S11), suggesting that these dinoflagellates were behaving as trophic specialists at the time of sampling despite their presumed capacity for mixotrophy. A similar phenomenon was observed with the five environmental prymnesiophyte bins, four of which also have the capacity for mixotrophy based on laboratory studies (44, 45). *Chrysochromulina brevifilum* was the only haptophyte species predominantly classified as a mixotroph; all other prymnesiophytes were predicted to have behaved primarily as trophic specialists at the time of sampling. Transcriptomes from two environmental dinoflagellate bins—the mixotrophic *Scrippsiella trochoidea* and the heterotrophic *Oxyrrhis marina*—resulted in conflicting predictions of phototrophy and heterotrophy (>25% for both categories across all classified transcriptional profiles) while lacking mixotrophic predictions. This suggested that the transcriptional profiles for these two taxonomic bins lie close to model decision boundaries (*SI Appendix*, *Investigating factors that may impact prediction quality for environmental transcriptome bins*) and, according to defined criteria, were not considered further (∼12% of the total retrieved transcriptomes; see *SI Appendix*, Fig. S12 for profiles including these bins). The stability of predictions over day/night cycles despite known diel oscillations in transcript abundances (46) further indicated that our model leverages a wide array of transcriptional features when predicting trophic mode. Overall, the predictions of heterotrophy dominated when the predictions were aggregated over each individual 24-h period (*n* = 4), and the prediction proportions remained stable over the diel period (Fig. 3*B*). Thus, this low-nutrient environment with abundant sunlight appeared to select for a predominance of heterotrophic feeding with less reliance on photosynthesis or mixotrophy, consistent with the hypothesis that phagotrophy provides protists with the necessary carbon and nutrients in oligotrophic environments.

To test whether species and community-level trophic modes changed under varying environmental conditions, we used SCOPE Gradients 1 (KOK1606, April/May 2016; *SI Appendix*, Fig. S10) metatranscriptome samples collected at a 15-m depth from 10 stations (see Dataset S12 for replication scheme) spanning from 23.5 to 37.3° N along 158° W from the oligotrophic subtropical gyre to the more nutrient-rich region of the North Pacific transition zone (*SI Appendix*, Fig. S10). The southernmost stations of Gradients 1 are within the gyre, ∼1° north of Station ALOHA, with similar community composition to that observed on the Diel cruise. A total of 47 metatranscriptomes (Dataset S12) were deconvolved into 437 transcriptional profiles representing 23 species-level taxonomic bins (Dataset S10 and *SI Appendix*, Fig. S11) distributed evenly between picoplankton (0.2 to 3 µm) and nanoplankton/microplankton (3 to 200 µm) size fractions, with ∼98% of the selected gene families present across the collection of retrieved profiles. Based on our required completeness and exclusion criteria (*SI Appendix*, *Investigating factors that may impact prediction quality for environmental transcriptome bins*), a total of 43 transcriptomes belonging to seven taxonomic bins were excluded from the large size fraction, and 23 transcriptomes belonging to four taxonomic bins were excluded from the small size fraction (removing a combined ∼15% of the retrieved transcriptomes). For both cruises, the exclusion of these bins did not affect community-level patterns (*SI Appendix*, Fig. S12). Of the 23 recovered taxonomic bins, 15 were also present in the Diel dataset.

The proportion of heterotrophic predictions was significantly reduced with increasing latitude (*P* ≪ 0.01; Fig. 3*C*). In the large size fraction, this was accompanied by an increase in predictions of mixotrophy. In the small size class, both mixotrophy and phototrophy predictions increased northward (Dataset S13). Both size fractions contained a core group of organisms present throughout the transect (*SI Appendix*, Fig. S13), with a reduced number of organisms found only at higher latitudes. We evaluated trophic mode predictions across the transect for three taxonomic species bins broadly distributed across sites—the mixotrophic species *Chrysochromulina rotalis*, *C. brevifilum*, and *Micromonas pusilla*. Within the gyre (demarcated at ∼30.5° N by the salinity front; *SI Appendix*, Fig. S10), the two *Chrysochromulina* sp. bins displayed contrasting strategies, with *C. rotalis* predicted to be primarily heterotrophic and *C. brevifilum* predicted to be mixotrophic (Fig. 3*D*) in agreement with the predictions for these same environmental species sampled during the Diel cruise in the summer of the previous year (Fig. 3*A*). Within the more nutrient-rich waters of the transition zone, we observed a shift to a greater reliance on photosynthesis, reflected in mixotrophy predictions for *C. rotalis* and increasingly split predictions between phototrophy and mixotrophy for *C. brevifilum* at higher latitudes. Although the latitudinal range of *M. pusilla* was not as broad, we also observed a distinct shift in trophic mode predictions from mixotrophy to phototrophy at higher latitudes. These results support the hypothesis that nutrient availability is an important driver of the trophic mode employed by these flexible organisms in the surface waters of the North Pacific.

### Environmental Drivers of Planktonic Nutritional Strategies.

**Fig. 4.**
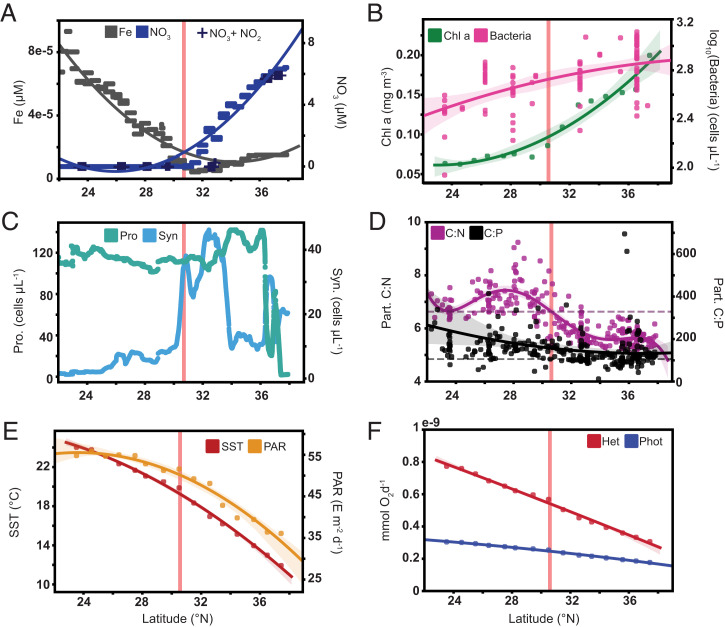
Environmental drivers of trophic mode in Gradients 1 surface waters. (*A*) Counter gradients in PISCES-v2 modeled Fe (gray) and NO_3_ (blue) or measured nitrate + nitrite (+). (*B*) Moderate Resolution Imaging Spectroradiometer satellite-derived chlorophyll *a* concentration (*SI Appendix*, *Methods*) and flow cytometry-based heterotrophic bacterial counts. Solid lines (*A* and *B*) represent second order fits to the data and shaded regions the 95% CI. (*C*) *Synechococcus* abundance and *Prochlorococcus* abundance derived from the shipboard flow cytometer SeaFlow. (*D*) Particulate C:N and C:P. The horizontal dashed vertical line indicates canonical Redfield ratios (C:N: purple; C:P: black) Solid lines represent fifth order (C:N) or second order (C:P) fits to the data and shaded regions the 95% CIs. (*E*) PAR and sea surface temperature derived from satellite products (73, 74). Solid lines represent a second order fit to the data and the shaded region the 95% CI. (*F*) Derived metabolic scaling relationships predict a disproportionate temperature-driven decrease in respiration rates for heterotrophs with increasing latitude. Solid lines are a linear fit of the data, shaded regions indicate 95% CI. See *SI Appendix*, *Calculation of respiration rates via metabolic theory* for details on metabolic rate calculations. Vertical red lines (*A–F*) indicate location of estimated salinity front near 30°N.

Resource allocation models (24) predict that the ratio of prey to nutrient availability determines trophic status. The Gradients 1 cruise spanned gradients in inorganic nutrient concentrations, microbial biomass, surface ocean temperature, and surface light levels (photosynthetically available radiation or PAR) (Fig. 4). Available nitrate, photosynthetic (satellite-derived), and bacterial (measured) biomass increased northward, with particulate carbon:nitrogen (C:N) and carbon:phosphorus (C:P) ratios that transitioned from higher than the Redfield ratio [106 C:16 N:1 P (47)] within the subtropical gyre (48) (<∼30.5° N) to below (C:N) or to (C:P) the Redfield ratio within the transition zone. The northward increase in biomass and productivity occurred against a backdrop of decreasing sea surface temperature, light, and iron availability. Heterotrophic predictions decreased in proportion northward with decreasing sea surface temperature and increasing prey availability. This result is consistent with the metabolic theory of ecology (MTE), which predicts a disproportionate decrease in respiration rates for heterotrophic organisms, given the light and temperature fields along the Gradients 1 transect (Fig. 4*F*). Phototrophic (0.2 to 3 μm) and mixotrophic prediction proportions increased northward with increasing dissolved nitrate, decreasing particulate C:N, and decreasing PAR. Our results suggest that a combination of temperature dependencies and quality of prey items (i.e., particulate C:N for nitrogen-limited phagotrophs) influences whether mixotrophic organisms rely primarily on nutrients derived from prey via phagotrophy or on dissolved nutrients and photosynthesis, a finding consistent with optimal foraging theory (OFT) (49, 50). Thus, nitrogen limitation in the subtropical gyre appears to drive mixotrophic organisms to rely primarily on phagotrophy to meet nitrogen and organic carbon demands, whereas increased nutrient availability at higher latitudes supports photosynthesis by mixotrophic organisms, potentially offsetting the increased costs associated with continued phagotrophy as temperature decreases.

**Fig. 5.**
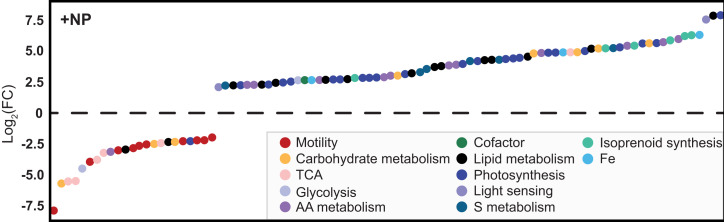
Transcriptional response of the subtropical gyre community of mixotrophic prymnesiophytes to nitrogen and phosphorous amendment. Bottle incubations were performed on the Gradients 2 cruise (MGL1704, May 26 to June 13, 2017) with cells harvested for RNA extraction before incubation and 96 h after amendment with 0.5 µM PO_4_ and 5 µM NO_3_ (*SI Appendix*, *Methods* and *Differential transcription analysis*). Significantly differentially transcribed genes |Log_2_(FC)| > 2 and *p_adj_* < 0.05 (224 total transcripts; Dataset S11) are indicated by circles colored according to PFAM classification and ordered by increasing Log_2_(FC).

We tested whether increased dissolved nutrient availability could cause mixotrophic organisms to shift their dominant trophic mode by analyzing metatranscriptomes from on-deck incubation experiments carried out on the SCOPE Gradients 2 cruise (MGL1704; May/June 2017; *SI Appendix*, Fig. S10). The addition of nitrogen and phosphorus to the nitrogen-limited subtropical gyre community allowed us to decouple shifts in nutrient availability from community compositional changes and shifts in environmental covariates along the cruise transect. The samples were collected before (t = 0) and after (t = 96 h) nutrient amendments. Logistical constraints resulting in smaller sample sizes meant that the metatranscriptomic data were deconvolved into mixotrophic prymnesiophyte environmental bins rather than individual species bins as required for trophic mode prediction. Instead, we analyzed these samples for patterns of differential gene expression. An amendment of samples with 5 µM nitrate and 0.5 µM phosphate led to significant (*p_adj_* < 0.05; *SI Appendix*, *Differential transcription analysis*) differential increases in transcript abundances (relative to t = 0) for genes encoding photosynthesis-related proteins and significant differential decreases in transcripts encoding proteins involved in cell motility (Fig. 5 and *SI Appendix*, *Methods* and *SI Appendix*, *Differential transcription analysis*). Other highly up-regulated transcripts were associated with amino acid metabolism, cytochromes, iron–sulfur enzymes, and lipid metabolism, indicating a significant restructuring of cellular metabolism and structure (Dataset S14). About a third of the significantly differentially transcribed genes (with |log_2_(FC)| > 2) were present in the combined set of selected features. The significant up-regulation of genes associated with phototrophy in our analysis of MMETSP transcriptomes and down-regulation of those related to heterotrophy suggest that mixotrophs such as prymnesiophytes can rapidly remodel their metabolism and shift their trophic strategy from phagotrophy to phototrophy in response to greater availability of limiting nutrients. We did not observe a similar shift in prymnesiophyte transcriptional patterns when nutrients were added to samples derived from the more nitrogen-rich, higher latitude waters of the transition zone (Dataset S15). Instead, additions of iron or a combination of iron, nitrate, and phosphate to this community resulted in a restructuring of the photosynthetic machinery and included a significant decrease (*p_adj_* < 0.05) in transcripts encoding the iron-responsive protein flavodoxin (51) and a significant increase in transcripts encoding plastocyanin (44). Together, these results highlight the remarkable ability of mixotrophs to rapidly optimize their nutritional strategy to environmental conditions.

## Discussion

Our study introduces a machine learning approach that leverages transcriptional profiles to predict the in situ trophic mode (heterotrophy, phototrophy, or mixotrophy) of protists in the natural environment. This method avoids potential artifacts associated with traditional, labor-intensive, incubation-based estimates and opens the door for large-scale studies of how carbon flows through specific members of microbial communities. The model was trained with transcriptomes derived from protists grown under controlled laboratory conditions and was challenged with a variety of validation transcriptomes. This approach identified a subset of gene family transcriptional profiles that, given sufficient transcriptome replicates, resulted in trophic mode predictions consistent with observed species-level nutritional strategies and broad phylogenetic patterns. The model accurately and consistently predicted the trophic mode of heterotrophic or phototrophic specialists, whereas mixotrophy predictions were often coupled with those of either heterotrophy or phototrophy, likely reflecting both overlapping and distinctive mixotrophic attributes. In addition, distinctive subsets of gene family clusters were identified that are associated with different trophic modes and can serve as starting points for understanding the molecular underpinnings of different metabolic strategies. A large public collection of microeukaryote transcriptomes (MMETSP) was used as the foundation for this work. However, the MMETSP was not designed for this particular application, and with more targeted molecular studies on mixotrophic organisms to include in the training set, the predictive power of this model will be improved.

We predicted significant shifts in the trophic mode of natural communities under different environmental conditions both at a community level and for taxonomic bins corresponding to individual populations. Within the oligotrophic gyre, our model predicts that protists rely primarily on phagotrophy to acquire sufficient carbon and nitrogen for growth, a result consistent with OFT, a resource allocation model (24), and a global ecosystem model that incorporates mixotrophy (29). Observed biomass C:N ratios greater than the Redfield ratio and results from our on-deck transcriptomics experiments suggest that, under nitrogen limitation, mixotrophs prioritize phagotrophy to acquire nitrogen from their prey (Fig. 5). The lower intracellular C:N and C:P ratios of marine prokaryotes compared with protists (45) makes phagotrophy even better suited to fulfill the nutrient requirements of cells in the nitrogen-limited waters of the gyre. Given the presumed optimality of heterotrophic metabolism, why do mixotrophic organisms with both phagotrophic and photosynthetic capabilities dominate protist communities within the oligotrophic gyre? We propose that, similar to what was observed in the amendment experiments, ephemeral injections of nitrogen into surface waters (52, 53) rapidly shift the mixotrophic community to more energetically favorable photosynthesis-based growth, thus preventing their displacement by heterotrophic specialists. During the dominant low-nutrient conditions, the ability of mixotrophs to consume prey (including photosynthetic prey) to fulfill their carbon and nitrogen requirements prevents their displacement by protist phototrophic specialists. The variable conditions of the gyre thus appear to mitigate potential costs associated with maintaining the complicated cellular machinery required for both photosynthesis and phagotrophy.

In the nutrient-rich North Pacific transition zone, a different scenario likely regulates the trophic modes of the protist community. As nutrient concentrations increase, our model predicts that the community shifts from a primary reliance on phagotrophy in the gyre toward an increasing reliance on phototrophy and mixotrophy by smaller protists (0.2 to 3 µm) and mixotrophy by larger protists (3 to 200 µm). A similar shift with latitude is predicted for individual species, with increased predictions of phototrophy for the small chlorophyte *Micromonas* and mixotrophy for the larger *Chrysochromulina* sp. The differing proportions of phototrophy predictions between size classes likely reflects the relationship between size-dependent uptake kinetics and nutrient availability, as smaller cells with a greater surface area:volume can more efficiently capitalize on lower dissolved nutrient concentrations (54). However, the MTE predicts rates of heterotrophic metabolism will decrease disproportionately with decreasing temperature (55, 56). Why, then, do the larger mixotrophs continue to carry out phagotrophy in the cooler, nutrient-rich waters of the transition zone? Recent studies suggest that mixotrophic cells use the reductants generated via photosynthesis for organic carbon metabolism rather than carbon fixation (57). Moreover, the mixotrophs within our training set were distinguished by the transcription of gene families encoding distinctive varieties of carbohydrate active enzymes. Thus, we propose that, at the highest latitudes reached on the Gradients cruise, these larger mixotrophs fulfill their nutrient requirements via prey engulfment despite reduced sea surface temperatures with the necessary reductants for organic carbon metabolism supplemented through photosynthesis. We further propose that, in accordance with the results from the Edwards model (24), the reduced light levels we observed at the higher latitudes remain sufficient to have little impact on mixotroph abundance in surface waters.

Deciphering the functional role of mixotrophic protists in the marine carbon cycle has been a longstanding challenge, made difficult by the many caveats involved with current methods. Here, we introduced a machine learning approach, made possible by the recent explosion of available transcriptomic data, and demonstrated that the model is skilled at inferring the trophic mode of natural populations based on the transcriptional patterns of select gene families. When we applied the model to metatranscriptomic data from the open ocean, the predicted patterns in trophic mode compare favorably with other results based on resource allocation models (24), global ecosystem models (29), the MTE (58), and OFT (49). By combining model predictions with the bioinformatic analysis of metatranscriptomes obtained during on-board incubation experiments, we were able to develop intuitive explanations for observed functional differences between organisms and highlight the potential drivers of mixotroph ecosystem function. As the ubiquity of mixotrophy in the marine environment becomes increasingly apparent, so does the need to incorporate mixotrophs into our understanding of the ocean’s carbon cycle and the microbial ecology of the marine water column. The future coupling of our machine learning technique with targeted field experiments and numerical modeling will enable detailed dissection of the role that mixotrophs play in marine ecosystem processes.

## Methods

### Model Training and Evaluation.

The MMETSP represents an imbalanced dataset for classification purposes (275 phototrophic, 93 mixotrophic, 78 heterotrophic, and remainder unknown). We therefore carried out feature selection using four datasets consisting of randomly under-sampled phototrophic transcriptomes (*n* = 80, 100, 120, and 140) together with all mixotrophic and heterotrophic transcriptomes. The features that impacted classification accuracy were determined for the Random Forest and XGBoost classifiers using an in-house version of the train-test MDA method (59). In our implementation of the MDA, the reduction in accuracy for a model is determined for each feature by randomly shuffling each feature across samples and carrying out fivefold cross-validation. The features that resulted in a decrease in classification accuracy were retained. To examine the clustered median expression of selected features in labeled MMETSP transcriptomes, data were reduced to the set of selected features, grouped by trophic mode, genes with zero median transcript abundances removed, and values were log transformed. T-SNE was carried out on reduced data to visualize the impact of feature selection on the separation between trophic modes in the t-SNE latent space. T-SNE was carried out with a high perplexity (100) in order to emphasize global relationships between the reduced transcriptomes (60). After feature selection, model performance was re-evaluated against the two reduced datasets (combined and common set). The list of selected Pfams is available in Dataset S3. The tree-based algorithms of Random Forest (90 ± 8%) and XGBoost (88 ± 10%) were significantly more precise than an artificial neural network (77 ± 6%) (Kruskal–Wallis H test; post-hoc Wilcoxon rank sum test; *P* < 0.05), and metric values increased when reduced feature sets were used during model training (*SI Appendix*, Fig. S14). Statistical measures were computed via the Kruskal–Wallis H test with a post-hoc pairwise Wilcoxon rank sum test with the Benjamini–Hochberg correction for multiple comparisons.

### Validation Transcriptome Processing.

The validation transcriptomes (Dataset S9) were retrieved from National Center for Biotechnology Information’s short read archive (SRA) through the SRA Toolkit and processed using an assembly and annotation pipeline similar to that presented in Johnson et al. (2018). Briefly, the reads were quality controlled using Trimmomatic (v0.36) (61) and normalized using the normalize-by-median.py script from the khmer software package (62). Normalized reads were assembled with Trinity (v2.9.1) (63) and annotated via the dammit! pipeline (v1.2; https://github.com/dib-lab/dammit). Bowtie2 (64) was used to assess the percentage of quality-controlled paired-end reads that mapped back to the assemblies. For dammit! annotations, only matches to the Pfam database with an e-value < 10^−5^ were retained. The read counts in transcripts per million were generated for each assembly using Salmon (v1.2.1) in quasi-mapping mode.

### Metatranscriptomic Data Processing.

The environmental transcriptome bins were obtained as follows: quality-controlled short reads were assembled using the Trinity de novo transcriptome assembler version 2.3.2 (63) on the Pittsburgh Supercomputing Center’s Bridges Large Memory system. The parameters included using in silico normalization, a minimum k-mer coverage of 2, and a minimum contig length of 300 base pairs. The raw assemblies were quality controlled with Transrate v1.0.3 (65). The assemblies were merged and clustered at the 99% amino acid identity threshold level with linclust in the MMseqs2 package (66). The translated contigs were aligned to a reference sequence database that included peptide sequences from hundreds of marine eukaryotic transcriptomes (67) using DIAMOND (double index alignment of next-generation sequencing data) v 0.9.18 (68). Taxonomy was assigned with DIAMOND by using the top 10% of hits with e-value scores below 10^−5^ to establish the lowest common ancestor of each contig. The putative function was assigned using hmmsearch [from HMMER 3.1b2 (69) using given trusted cutoff bitscores, –cut_tc] to find the best-scoring gene family from Pfam 31.0 (32). The contig abundances were quantified by the pseudoalignment of the paired reads to the assemblies with kallisto (70) and normalized to the total assigned read pool of the taxonomic bin. Species-level transcriptional profiles were normalized in silico to generate transcripts per million profiles. Bin completeness was estimated by the number of nonzero transcript abundances within each bin. A completeness cutoff of 800 nonzero transcripts was selected based on the distribution present in the MMETSP dataset (*SI Appendix*, *Investigating factors that may impact prediction quality for environmental transcriptome bins*). Detailed information concerning RNA extraction and library preparation can be found in *SI Appendix*, *Methods*.

### Environmental Metadata Sourcing and Processing.

The environmental metadata and cruise data were obtained from the Simons Collaborative Marine Atlas Project pycmap API (71) (https://simonscmap.com/; data originating from refs. 72 to 76). The respiration rates were calculated following the equations presented in ref. 77 using satellite-derived sea surface temperature (75) and PAR (74) as input. The details of these rate calculations are presented in *SI Appendix*, *Calculation of respiration rates via metabolic theory*. A detailed discussion concerning the origin and preprocessing of ancillary environmental metadata may be found in *SI Appendix*, *Methods*.

### At-Sea Incubation Experiments.

A total of 20 L seawater was collected into replicate polycarbonate carboys from 15-m depth and incubated at in situ temperature for 96 h in on-deck, temperature-controlled incubators screened with 1/8-in light blue acrylic panels to approximate in situ light levels at 15 m [55% of surface irradiance to approximate in situ light levels assuming an attenuation coefficient of 0.04 m^−1^ (78)]. Triplicate carboys were amended with 5 µM nitrate and 0.5 µM phosphate at *t* = 0. Duplicate carboys with no amendment served as a control. After 96 h, the samples were filtered onto a 3-µm polycarbonate filter. The cells passing through the 3-µm filter were collected on a 0.2-µm polycarbonate filter. RNA extraction and sequencing were carried out as detailed in *SI Appendix*, *Methods*. The metatranscriptome reads from shipboard incubation experiments were mapped to prymnesiophyte reference transcriptomes using Salmon (79). The number of reads mapped from all treatments was consistently between 300,000 to 400,000. Differentially transcribed genes were identified with R using DESeq2 (80) with an adjusted *P* value cutoff of 0.05. A detailed discussion of this analysis is presented in *SI Appendix*, *Differential transcription analysis*.

## Supplementary Material

Supplementary File

Supplementary File

Supplementary File

Supplementary File

Supplementary File

Supplementary File

Supplementary File

Supplementary File

Supplementary File

Supplementary File

Supplementary File

Supplementary File

Supplementary File

Supplementary File

Supplementary File

Supplementary File

Supplementary File

## Data Availability

The scripts used for feature selection, model training, and prediction are available in GitHub at http://www.github.com/armbrustlab/trophic-mode-ml. The training data are available through Zenodo (DOI: 10.5281/zenodo.4425690) (81) . The metatranscriptome data from the SCOPE Diel cruise (KM1513) are available through the NCBI’s SRA under BioProject PRJNA492142, SCOPE Gradients 1 under BioProject PRJNA690573, and SCOPE Gradients 2 incubation experiments under BioProject PRJNA690575. *Chrysochromulina* sp. isolate transcriptomes are available under BioProject PRJNA690570. For ancillary environmental data accession information, please see Dataset S16.
