# Supplementary material for: The dynamic trophic architecture of open-ocean protist communities revealed through machine-guided metatranscriptomics
Source: Proc Natl Acad Sci U S A. 2022 Feb 10;119(7):e2100916119. doi: 10.1073/pnas.2100916119 (PMC8851463; doi:10.1073/pnas.2100916119)
Supplement: Supplementary File [file pnas.2100916119.sapp.pdf]

Supplemental Information

Bennett S. Lambert[1,*], Ryan D. Groussman[1], Megan J. Schatz[1], Sacha N. Coesel[1], Bryndan P. Durham[2], Andrew J. Alverson[3], Angelicque E. White[4], E. Virginia Armbrust[1]

Author Affiliations:

1. School of Oceanography, University of Washington, 1501 NE Boat St., 98195, Seattle, WA, USA.
2. Department of Biology, University of Florida, 876 Newell Dr., 32611, Gainesville, FL, USA.
3. Department of Biological Sciences, University of Arkansas, 850 W Dickson St., 72701, Fayetteville, AR, USA.
4. Department of Oceanography, University of Hawai'i at Manoa, 1000 Pope Rd., 96822, Honolulu, HI, USA.

*Corresponding author: lambertb@uw.edu

Supplemental Information: Methods

Training data sources and processing

Transcriptomes for model training were obtained from the Johnson et al. MMETSP re-assemblies (1). Salmon (2) files, combined with dammit! (v1.2; https://github.com/dib-lab/dammit) annotations, from the re-assemblies were used to generate Pfam (3) transcriptional profiles in transcripts per million (TPM). Each sample of the MMETSP was labelled 'mixotrophic', 'heterotrophic', 'phototrophic', or 'unknown' based on information from the literature (see Dataset S2) and associated metadata. The following conserved lineage-specific patterns were also included: All diatoms present in the MMETSP were labelled phototrophic, all chlorophytes were labelled phototrophic (unless contrary evidence in the literature existed), foraminifera were labelled heterotrophic, all amoeba were labelled heterotrophic, and all parasitic eukaryotes were labelled heterotrophic. All obligate osmoheterotrophic microeukaryotes were withheld from the training data, prior to model training. Samples were labelled mixotrophic if culture conditions were not axenic, light was supplied, and the organism had been previously reported to ingest particles.

Data pre-processing

Analysis was carried out in Python 3.7, using Pandas (v0.25.1), NumPy (v1.17.2), Scikit-learn (v0.21.3), XGBoost (v0.90), and Keras (v2.3.1) with Tensorflow (v2.0.0). All gene family transcriptional profiles were aggregated into a single table, with missing values filled using zero imputation. This process ensured that each sample's feature vector shared all gene families present throughout the MMETSP. The dataset was split into training and test sets (test_size = 0.33). Data were scaled between 0 and 1 using Scikit-learn's MinMaxScaler, fit solely on the training data, prior to model training and application.

Hyperparameter optimization

To select the best performing hyperparameters, a grid search was performed using the Scikit-learn method GridSearchCV with 5-fold cross-validation for each model examined in this study (Dataset S17). To reduce the potential for artefacts that may arise from carrying out parameter searches on an imbalanced dataset, the training data were subsampled and phototrophic transcriptomes were under-sampled (n = 100) to create a dataset with a more balanced class distribution. A parameter search was also carried out to determine the most performant ANN architecture (Fig. S3). Dropout rate, regularization, batch size, learning rate, and the number of hidden nodes were examined for their effect on the performance of the feed-forward network.

Model comparison

We evaluated whether use of an Artificial Neural Network (4) could increase the precision of trophic mode predictions for MMETSP organisms based on both the entire set of ~9000 gene families as well as the reduced sets of selected gene families (the union and common set of selected features). After model tuning, the greatest precision, recall, F1 score, and accuracy were achieved with Random Forest and XGBoost (Fig. S3). Random Forest (90 ± 8%) and XGBoost (88 ± 10%) were significantly more precise than the Artificial Neural Network (77 ± 6%) (Fig. S3; Kruskal-Wallis H test; post-hoc Wilcoxon rank sum test; $p < 0.05$), which displayed evidence of overfitting (5) during model training. The reduced feature sets increased mean metric values for both Random Forest and XGBoost (hatched and outlined bars, Fig. S3), both of which consistently outperformed the Feed-Forward Neural Network (and other simple neural networks) across all assessed metrics.

Phylogenetic tree construction

Representative 18S rRNA sequences were obtained from the National Center for Biotechnology Information nucleotide database. Sequences were aligned using MAFFT (v7.453) (6). Alignments were performed with the FFT-NS-2 method and a tree was constructed with RAxML (7) using the GTRGAMMA model with 25 bootstraps. All trees were visualized via the Interactive Tree of Life (8).

Cell culture conditions

Two isolates of *Chrysochromulina* sp. (KB-HA01, AL-TEMP) were obtained from G. Stewart (U. Hawaii). *Chrysochromulina* sp. cultures were maintained at 20 degrees C, on a 16:8 hr light/dark cycle, with approximately 100 μmol photons m^{-2} s^{-1} PAR, using cool white fluorescent lights. *Chrysochromulina* sp. (KB-HA01) was grown in f/2 media and *Chrysochromulina* sp. (AL-TEMP) was grown in K media.

Feeding experiments and microscopy

Chrysochromulina sp. (AL-TEMP and KB-HA01) cells were grown until late exponential phase (K and f/2 media, respectively) when 1 μm (Invitrogen) and 0.75 μm (Polysciences) fluorescent polystyrene beads (50/50 ratio) were added to a final concentration of approximately 10^5 mL^{-1}. Samples were incubated for 1 hr prior to image acquisition. The cell and bead solution was mounted on a microscope slide and images were acquired using a Leica DMi8 inverted microscope.

RNA extraction, library preparation, and sequencing

Diel sampling – Seawater samples were recovered from a 24 × 12 L Niskin bottle rosette attached to a conductivity-temperature-depth (CTD) package (SBE 911Plus, SeaBird). Diel samples were processed as described in Coesel et al. (2021). Briefly, samples were collected from ~15 m depth every 4 hrs over a 4-day period. Seawater was pre-filtered using 100 μm Nitex mesh and collected on a 0.2 μm

polycarbonate filter. Total RNA was extracted using the ToTALLY RNA kit (Invitrogen) and poly(A)-selected mRNAs were used for Illumina NextSeq 500 sequencing.

Gradients 1 station sampling – Seawater samples were collected from a 24 × 12 L Niskin bottle rosette attached to a conductivity-temperature-depth (CTD) package (SBE 911Plus, SeaBird). Samples were collected at 15m depth and pre-filtered through 200 μm Nitex mesh onto a 3 μm polycarbonate filter. Cells passing through the 3 μm filter were collected on a 0.2 μm polycarbonate filter, yielding 2 size classes (0.2 – 3 μm and 3 – 200 μm). Total RNA was extracted using the ToTALLY RNA kit (Invitrogen) and poly(A)-selected mRNAs were sequenced on the Illumina NextSeq 500 platform.

Gradients 2 incubation sampling – In situ seawater samples were collected as described for Gradients 1 with the exception that a 100 μm Nitex mesh was used as a pre-filter. Incubation samples were collected using a trace-metal clean bellows pump from 15 m depth, and after 96 hr, samples were filtered sequentially onto 3 μm and 0.2 μm polycarbonate filters. Total RNA was extracted using the Direct-zol RNA MiniPrep Plus kit (Zymo Research) and poly(A)-selected mRNAs were sequenced on the Illumina NovaSeq platform.

Chrysochromulina cultures – Exponentially growing cultures were filtered onto 47 mm polycarbonate filters 2 hrs prior to dark conditions and 2 hrs following the onset of darkness (12:12 light:dark cycle). Approximately 100 mL was filtered for each culture and condition. Total RNA was extracted using the Direct-zol RNA MiniPrep Plus kit (Zymo Research) and poly(A)-selected mRNAs were sequenced on the Illumina NextSeq platform.

For all samples extracted RNA was quantified using a Qubit fluorometer (Thermofisher) and quality controlled using a Bioanalyzer (Agilent) prior to sequencing. Samples were randomized across sequencing runs to reduce potential biases.

Classification of taxonomic bin transcriptional profiles

To classify field samples, each model was trained with the MinMax scaled MMETSP training dataset containing the union of selected features. Predictions were then generated for each retrieved transcriptional profile by subsetting each profile to contain the union of selected features, imputing missing values with zeros, and scaling profiles with the pre-fitted MinMax scaler. Models were then applied to processed transcriptional profiles to generate trophic mode predictions.

Determining environmental drivers of trophic mode

To assess how light/dark cycles impacted classification in the natural environment, Diel transcriptome bins were labelled as 'day' or 'night' samples prior to classification. Day samples were obtained at 10:00, 14:00, and 18:00, whereas night samples were obtained at 22:00, 02:00, and 06:00. The proportion of trophic mode predictions assigned to Diel transcriptome bins was then determined by aggregating predictions according to sample acquisition time. Taxonomic bins with prediction

proportions of greater than 25% in both phototrophy and heterotrophy were excluded (*n* = 2 bins; Note S1). Gradients 1 bins were grouped by latitude and replicate transcriptomes within a species bin were examined for prediction proportions of greater than 25% in both phototrophy and heterotrophy. When predictions for a taxonomic bin violated this criterion, all profiles for that bin were removed from that latitude. This filtering criteria led to the exclusion of 66 transcriptional profiles, belonging to 11 taxonomic bins.

Environmental metadata and cruise data were obtained using the Simons Collaborative Marine Atlas Project pycmap API (CMAP; https://simonscmap.com/). Data were co-localized with the Gradients 1 cruise using the cruise identifier KOK1606. Cruise trajectory plots presented in Fig. S9 were generated by retrieving satellite-derived sea surface salinity (9) from the 'tblSSS_NRT' table in CMAP via the Python API. Mean values for each latitude and longitude were computed over each cruise's duration. Cruise trajectory plots were generated using the Basemap Matplotlib toolkit (https://matplotlib.org/basemap) in Python 3.

PISCES-v2 (10) Iron, Nitrate, and Phosphate data were co-localized with the Gradients 1 cruise using a depth range of 0-30 m, a latitudinal and longitudinal tolerance of 0.25 degrees, and a temporal tolerance of 15 days. Particulate carbon, nitrogen, and phosphorus samples were collected from the ship's underway flow through system using a pair of semi-automated filtration systems (SAFS). Briefly, incoming seawater was routed through a flow meter (MacMillan Flo-Sensor Model 101) to a multi-position valve (Vici Cheminert, 10 position valve) programmed to rotate through a series of outlet ports connected to a GF/F filter holder (Swinney stainless steel 25 mm). Custom MATLAB code controlled valve position and recorded both flow rates and filtration time for each sample (~ 50-100 ml min^{-1}). At the end of each cycle filters were removed, stored in foil, labelled, and frozen at -20°C. Particulate C and N were analyzed on a CHNS analyzer (Carlo Erba, model NA1500) at the Oregon State University Stable Isotope Laboratory using cystine (29.99 % C and 11.66% N by weight) as the primary standard. Particulate P was analyzed via molybdenum blue spectrophotometry (11). *Prochlorococcus* and *Synechococcus* abundances were obtained through the ship-board flow cytometer SeaFlow (12, 13). Sea surface temperature was obtained from the Global Blended Sea Surface Temperature Analysis (14), a collection of satellite-derived estimates and *in situ* measurements (1/4 × 1/4° spatial resolution, daily average). Daily photosynthetically-available radiation (PAR) was retrieved from MODIS Aqua reprocessed data products (15) (9 × 9 km spatial resolution, daily average). *Chl* a was obtained from the Ocean Colour Thematic Center at the Copernicus Marine environment monitoring service (http://marine.copernicus.eu/) and consists of satellite-derived *Chl a* estimates across a series of satellites (25 × 25 km spatial resolution, 8 day average). All data above were retrieved from CMAP. Bacterial counts were obtained from samples collected during Gradients 1. Briefly, discrete samples were collected and amended with glutaraldehyde (0.2% final concentration),

incubated for 15-30 minutes in the dark, snap frozen and stored at -80 ºC until analysis. Heterotrophic bacteria were analyzed by staining each sample with SYBR Green (0.01% final concentration) for 15 minutes in the dark on ice. Stained samples were run on a BD Influx flow cytometer equipped with a small particle detector and cells were enumerated from a known volume of at least 100 µl. The samples were also analyzed unstained to enumerate picocyanobacteria. The concentration of picocyanobacteria was subtracted from the concentration of total bacteria to give the concentration of heterotrophic bacteria. Analyses were performed in triplicate.

Supplemental Figures

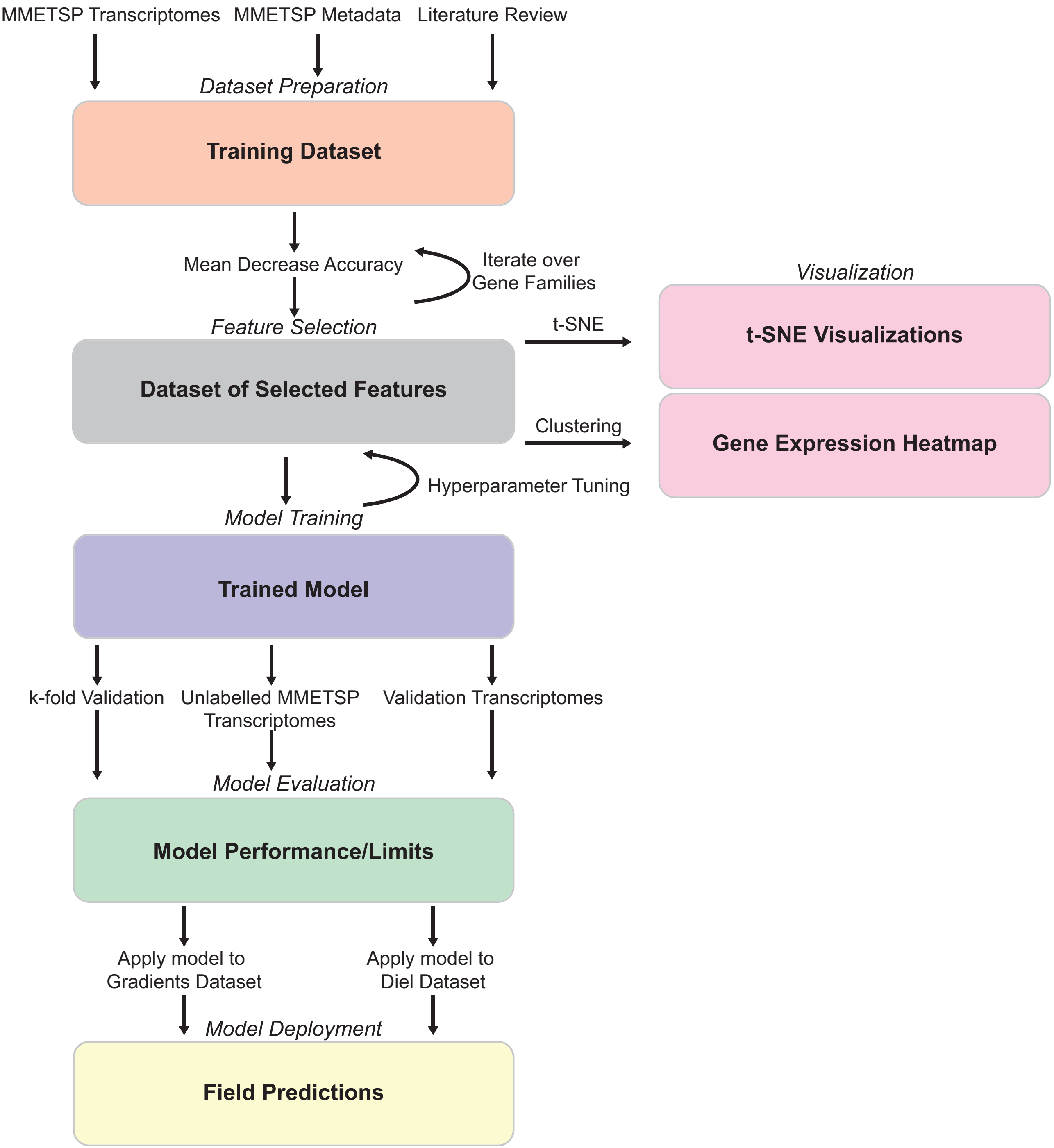

Figure S1. A flow chart illustrating data labelling process, feature selection, visualization, model training, evaluation, and application. Data from the MMETSP was labelled using associated metadata and a literature review. After labelling, gene families that most impact the model performance were selected through the Mean Decrease of Accuracy algorithm using Random Forest and XGBoost. The results of the feature selection process were visualized in two formats: t-sne to observe separation between trophic modes in reduced dimensions and a clustered heatmap of gene families to highlight specific gene family clusters that might drive separation of trophic modes in the selected gene family set. Hyperparameter tuning was performed to identify training parameter values for the two models investigated in this study. Models with optimized parameters were trained and evaluated through k-fold cross validation and applied to both unlabelled MMETSP transcriptomes and validation transcriptomes not present in the MMETSP. This process provided an overview of the expected performance of our model and constraints related to its application. Trained models were then used to make predictions for transcriptome bins retrieved from the Gradients 1 and Diel cruises.

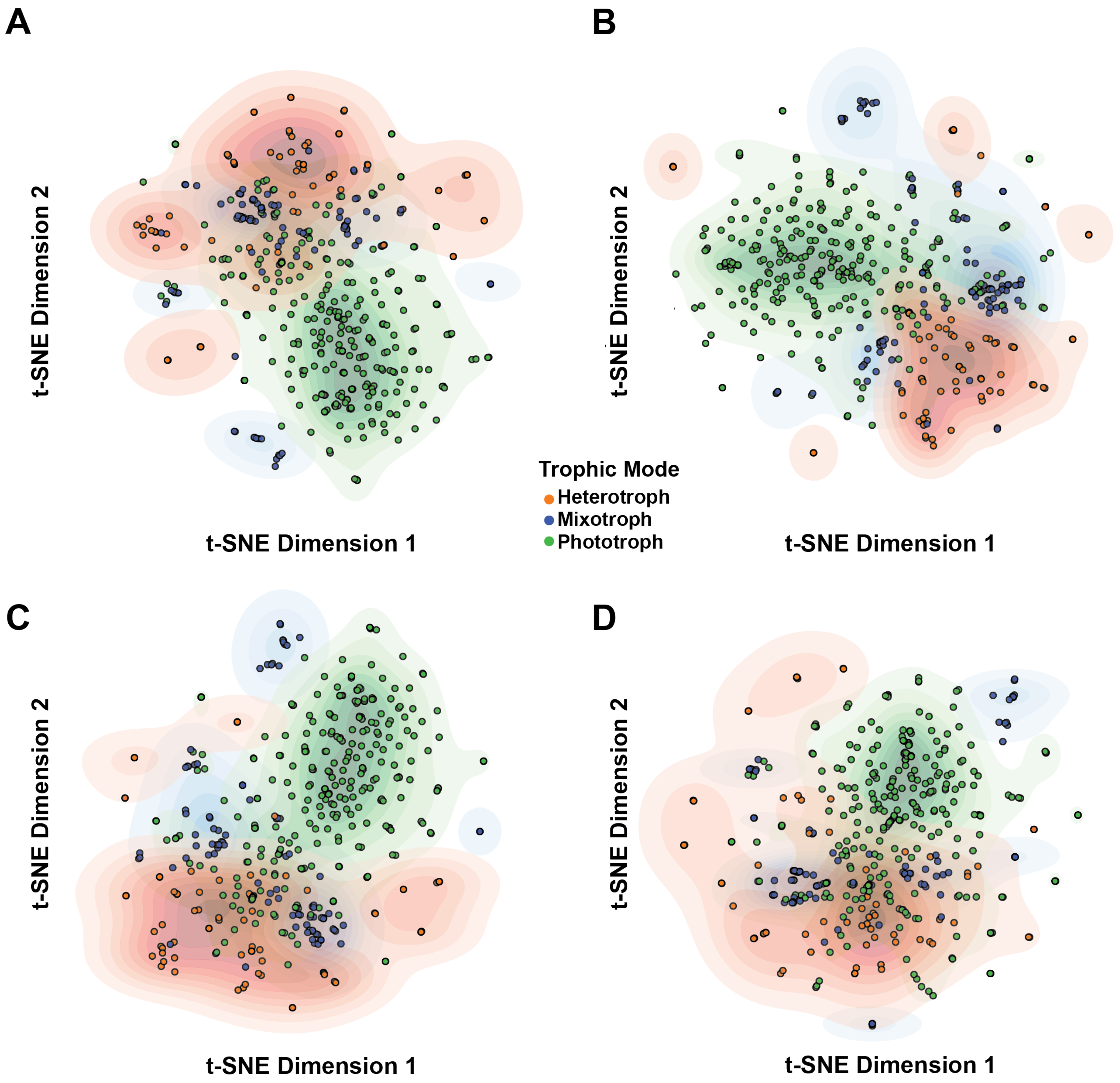

Figure S2. Enhanced separation of trophic modes through feature selection. T-SNE was applied to MMETSP transcriptomes using feature sets derived from (A) Random Forest ($f = 901$), (B) XGBoost ($f = 265$), and (C) the union of selected features ($f = 1046$). Transcriptional profiles containing selected features were MinMax scaled and t-SNE applied. Shading represents the kernel density estimate for each trophic mode. In each instance, phototrophs and heterotrophs are well separated in the resulting latent space. Mixotrophic transcriptomes are generally present as a bridge between the two specialist nutritional modes. (D) Separation between trophic modes was not as distinct when t-SNE was performed on training data with all transcripts present.

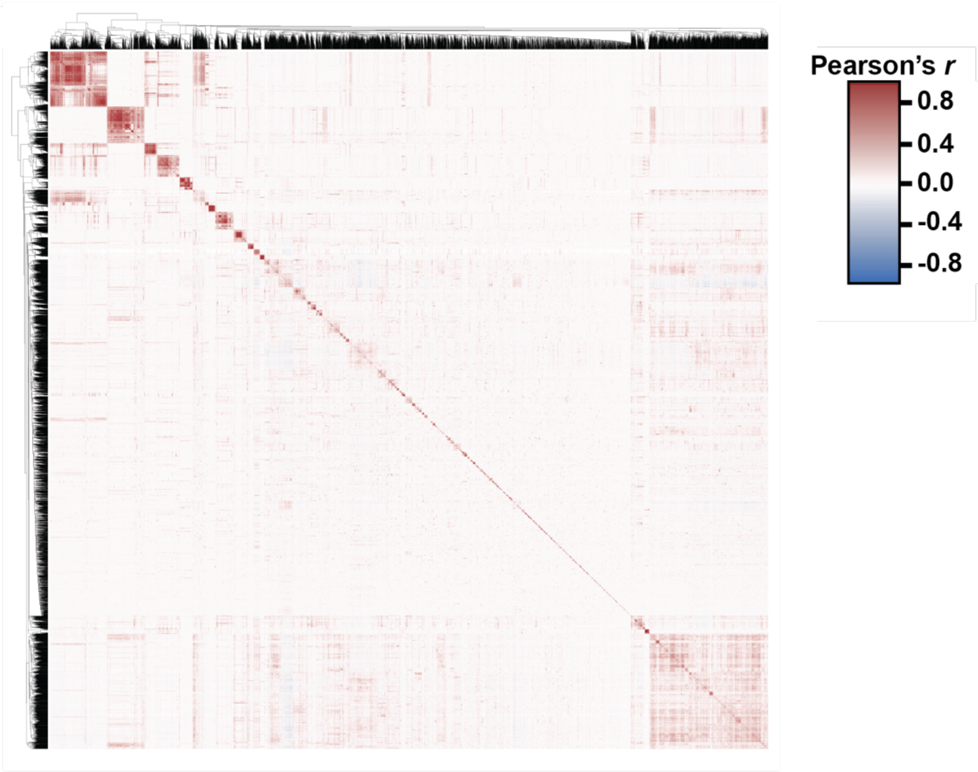

Figure S3. Correlations between gene families in the MMETSP training dataset. Datasets with strongly correlated features suffer from "multi-collinearity," which can impact predictive model performance. Over the entire MMETSP dataset, relatively few gene families are strongly correlated.

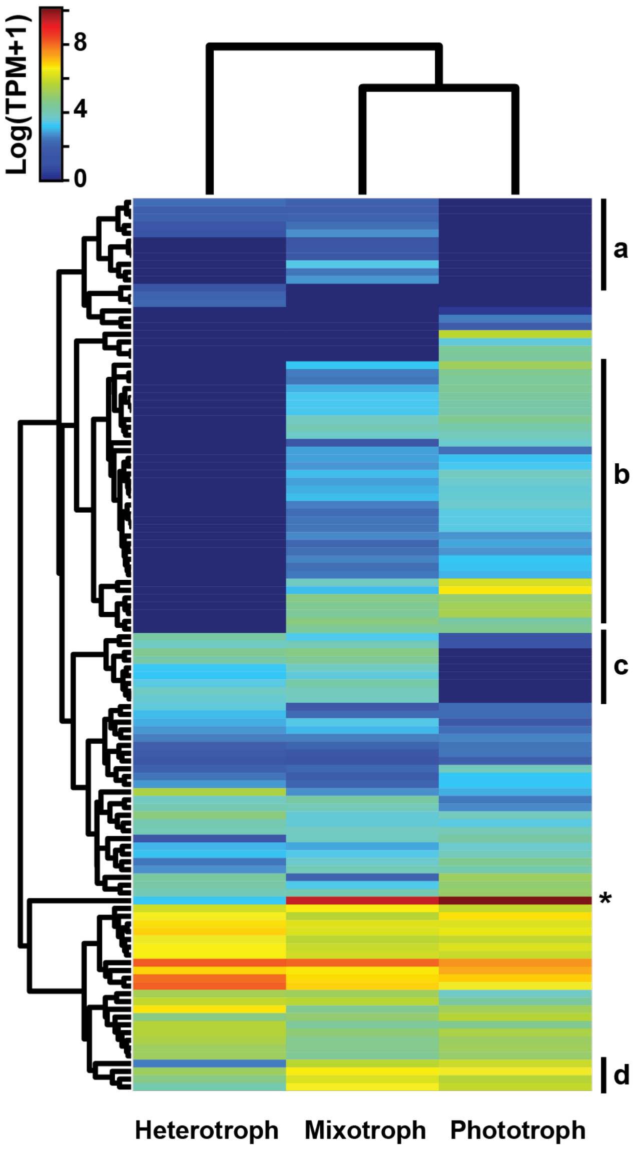

Figure S4. Median transcript abundance of common selected genes. Transcription of selected genes in the MMETSP highlights gene family clusters differentiating trophic modes, labelled on the right of the clustered heatmap (Dataset S6). Out of 120 common selected gene families, 115 had non-zero median expression in MMETSP transcriptomes. * Denotes a single transcript present in high abundance in both mixotrophic and phototrophic transcriptomes corresponding to a chlorophyll *a/b* binding protein.

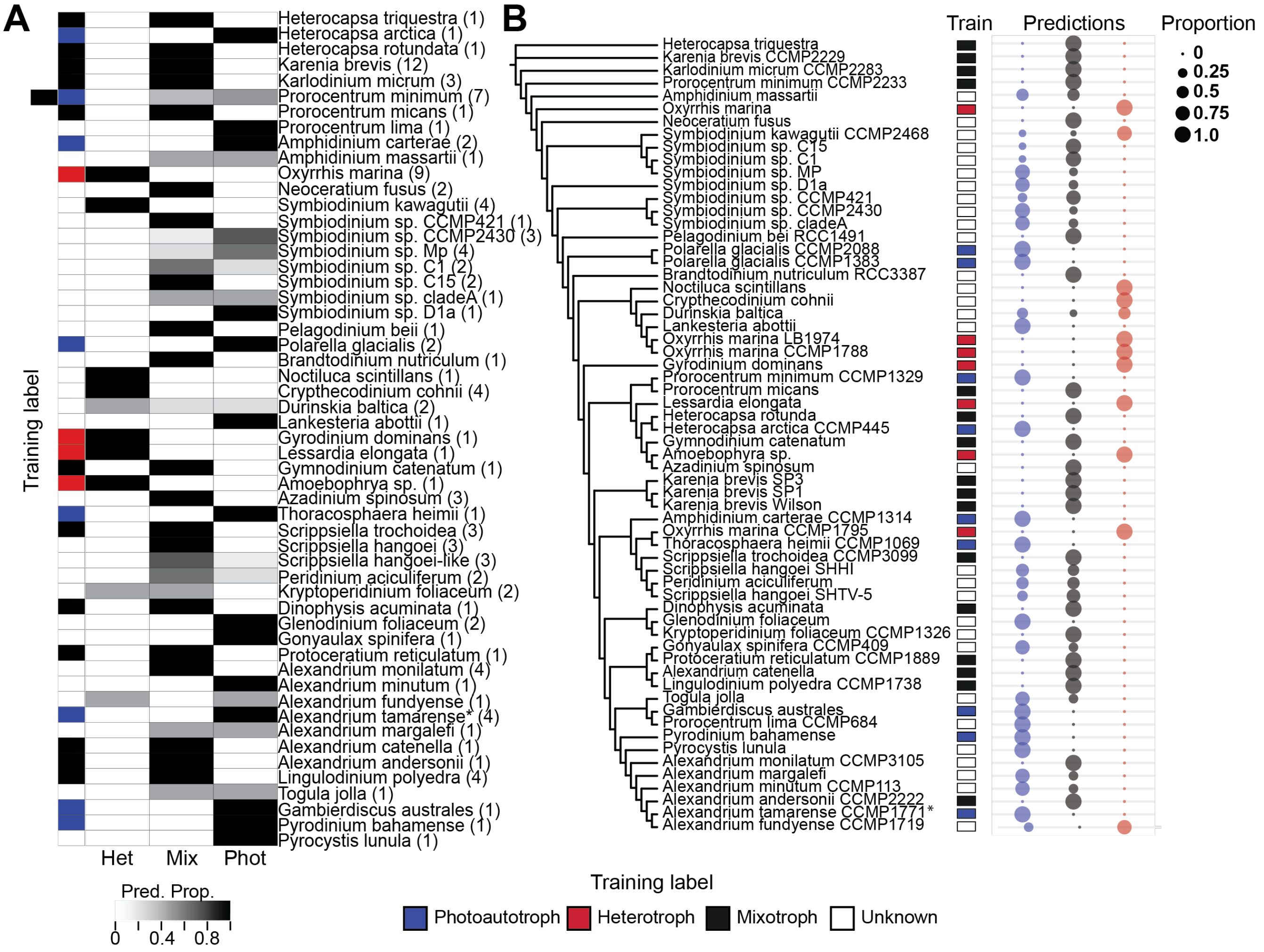

Figure S5: Predictions for unlabelled MMETSP Dinoflagellate transcriptomes. (A) Random Forest predictions. Indicated are the training labels, when available (annotation strip) and trophic mode predictions derived from Random Forest model output using both selected feature sets. **A. tamarense* was only grown axenically. The number of transcriptome replicates is displayed in parentheses. (B) Aggregated prediction proportions from both models (Random Forest, XGBoost) and both feature sets (union, common set) displayed as a bubble plot. Indicated are the training labels, when available (annotation strip). Bubble size indicates the proportion of predictions in each trophic mode class. Results are shown at the strain level.

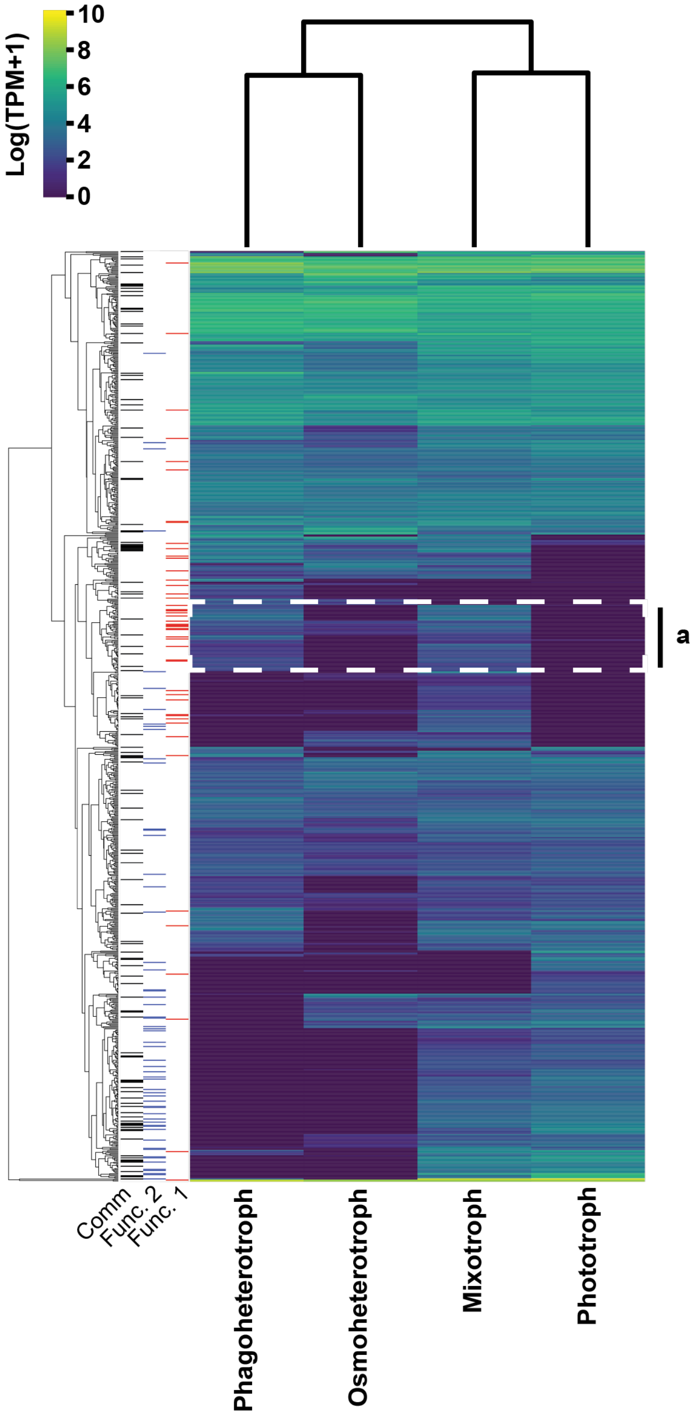

Figure S6. Median transcript abundance of the combined selected gene families (f =1046) including heterosmotrophic organisms. The cluster labelled "a" highlights genes transcribed by phagotrophs and mixotrophs but not osmoheterotrophs or phototrophs (Dataset S8).

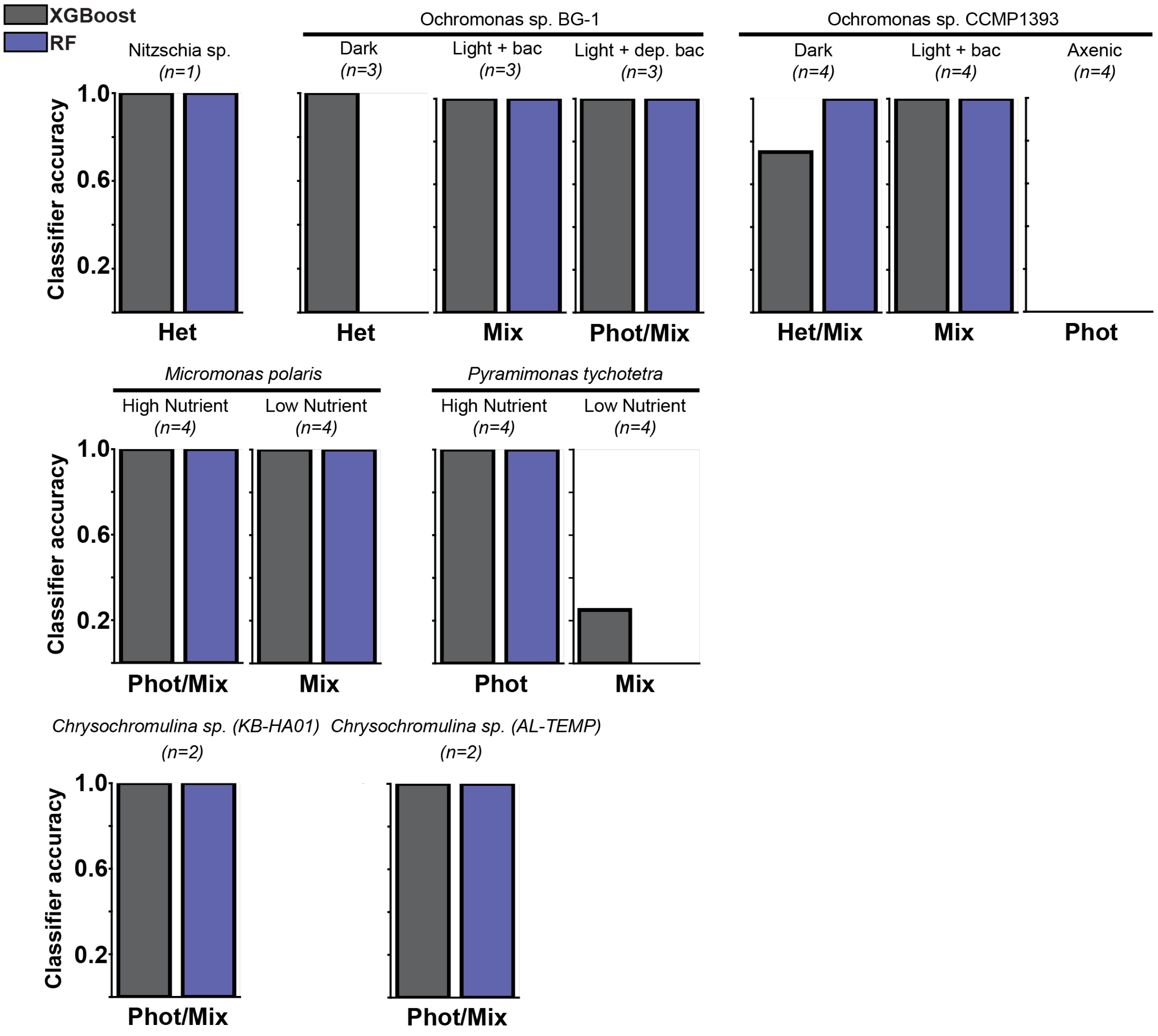

Figure S7. Performance of XGBoost and Random Forest classifiers on validation transcriptomes. Model performance was assessed through transcriptomes that were not present in the MMETSP. Transcriptomes were obtained from experiments carried out to drive mixotrophs into differing nutritional modes through manipulation of light, nutrient, and prey availability. XGBoost (gray) outperforms Random Forest (blue) when comparing prediction accuracy across all validation transcriptomes. *Nitzschia* sp. was accurately classified as heterotrophic despite no heterotrophic diatom transcriptomes and 4 closely related photosynthetic *Nitzschia* transcriptomes present in the training data. *Ochromonas* sp. BG-1 and CCMP1393 are represented in the MMETSP. Both classifiers failed to predict obligate phototrophy for *Ochromonas* sp. CCMP1393 grown in the absence of bacteria. Both models accurately identified mixotrophy in *Micromonas polaris*, where grazing was observed in both the high and low nutrient experimental conditions. The low nutrient condition, intended to induce mixotrophy in *Pyramimonas tychotetra*, challenged our models. Both models predicted phototrophy across both nutrient conditions. This could be due to the low rates of bacterial grazing observed under the low nutrient condition. Predictions for *Chrysochromulina* sp. (KB-HA01 and AL-TEMP) were consistent with observed particle ingestion in AL-TEMP and reports of grazing in numerous *Chrysochromulina* isolates. Expected prediction labels are present in bold beneath each plot.

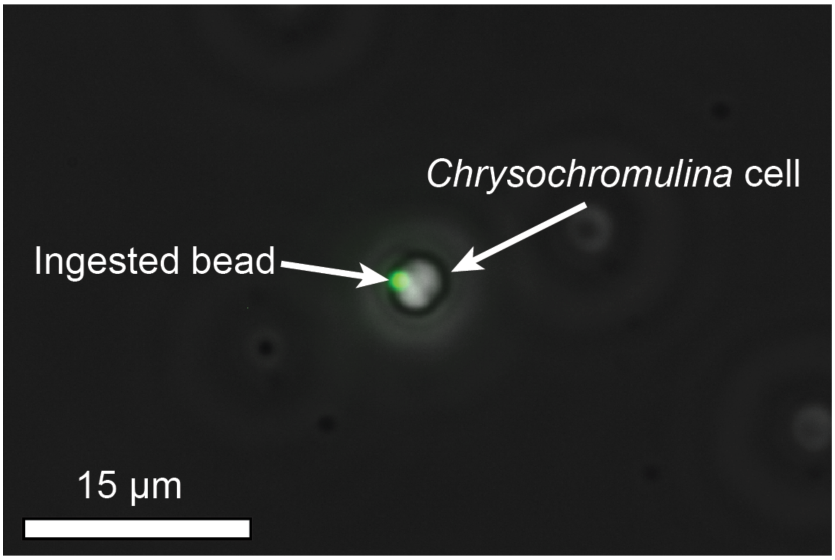

Figure S8. Particle ingestion by *Chrysochromulina* sp. (AL-TEMP) observed via microscopy. Cells were grown in K media until late exponential phase when fluorescent (0.75 and 1 μm; 50:50 ratio) polystyrene beads were added to an approximate concentration of 10^5 mL^{-1}. Samples were incubated for 1 hr prior to image acquisition.

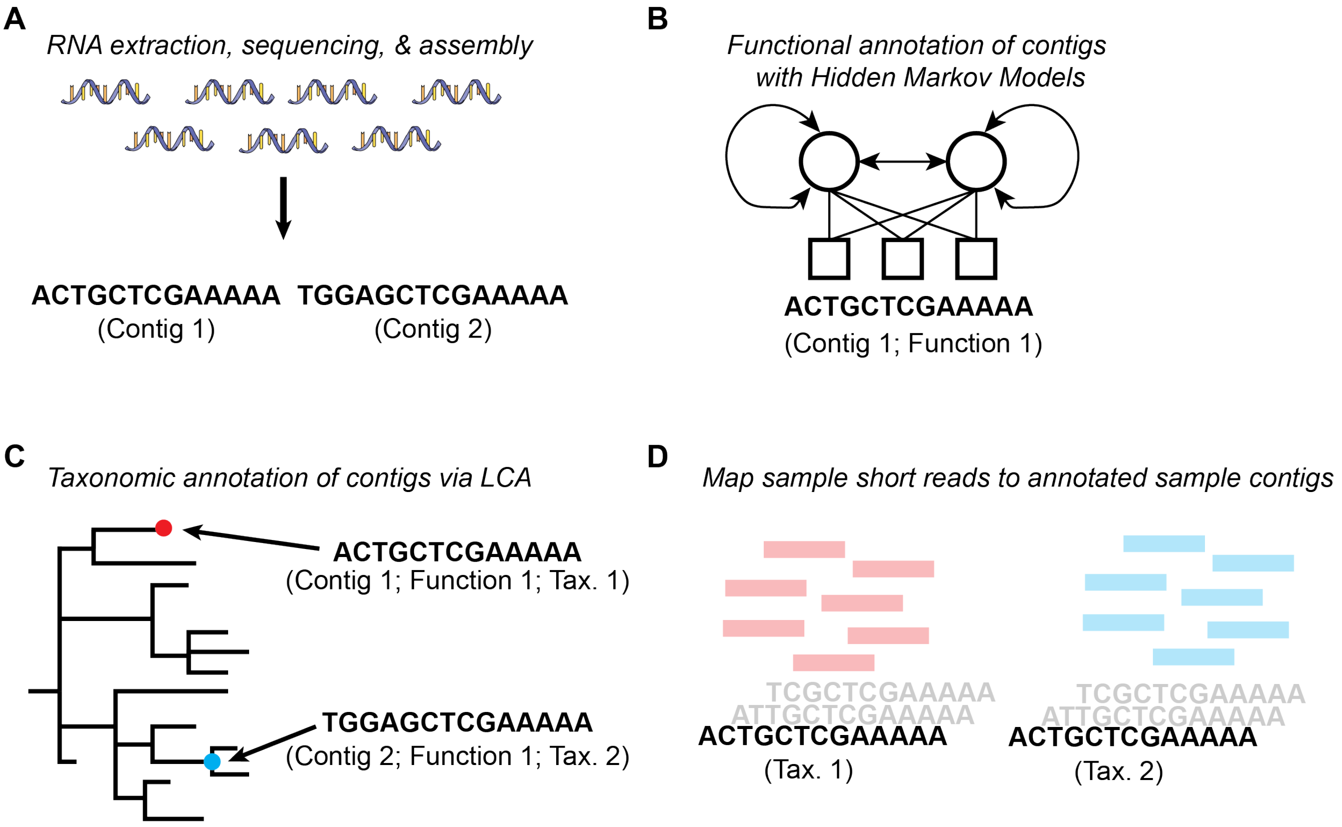

Figure S9: Overview of extraction of transcriptional profiles and taxonomic bins from metatranscriptomes. (A) RNA was extracted from filters corresponding to each cruise station and sequenced. Resulting short reads from replicates were co-assembled into longer contigs. (B) Each contig resulting from assemblies of replicate samples was assigned a putative function using a Hidden Markov Model-based approach (HMMER). (C) Each individual contig was assigned taxonomy using Lowest Common Ancestor (LCA; Diamond). During this process, a contig can be assigned a species-level annotation (red circle) or be annotated at a higher taxonomic level (blue circle) if sequences align with similar affinity (based on a match bit-score threshold) to multiple reference sequences present within the reference database. In general, conserved sequences receive annotations at higher taxonomic levels. Contigs assigned to the same taxonomy within the same sample are collected and considered a ‘taxonomic bin’. (D) To generate transcriptional profiles for each taxonomic bin, the short reads used to generate sample contigs were aligned to contigs and enumerated with kallisto to determine the transcript abundance of each contig in each taxonomic bin. See SI Methods for details of each step.

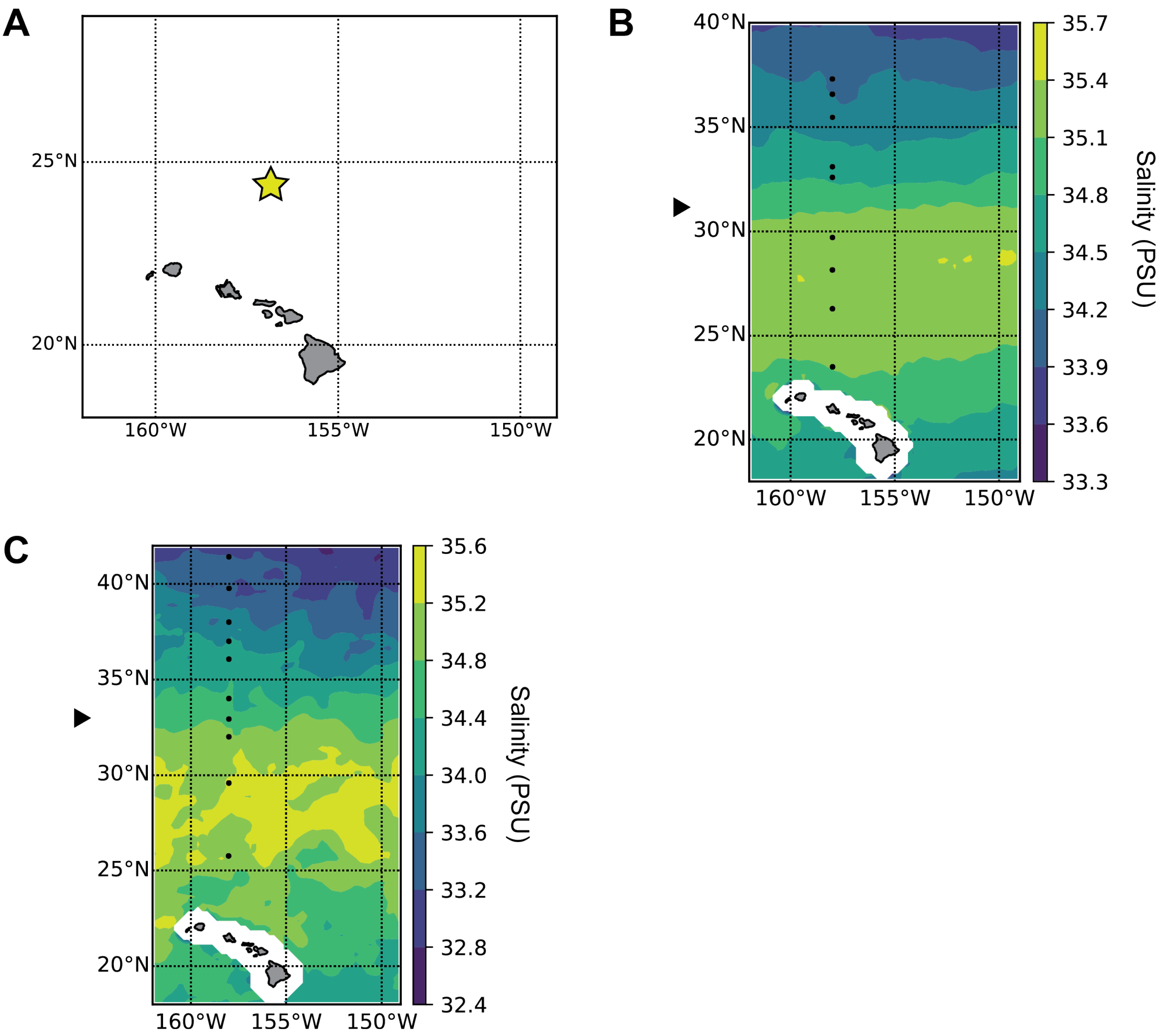

Figure S10. Field sites examined in this study (A) Diel (KM1513). A research cruise off the Hawaiian coast focused on biological activity over diel cycles. A water parcel was followed over a 4-day period by tracking a Lagrangian drifter. Samples were obtained for RNA extraction every 4 hours. (B) Gradients 1 (KOK1606). A transect crossing a region characterized by steep physical, chemical, and biological gradients in the North Pacific Ocean. The marker shows the approximate location of the salinity front that represents the boundary between the subtropical gyre and the transition zone. (C) Gradients 2 (MGL1704). A transect following and extending the trajectory of Gradients 1. The marker shows the approximate location of the salinity front that represents the boundary between the subtropical gyre and the transition zone. Average satellite-derived sea surface salinity (Remote Sensing Systems SMAP L2C Sea Surface Salinity, (9)) over the course of each cruise (B,C) is shown as background shading.

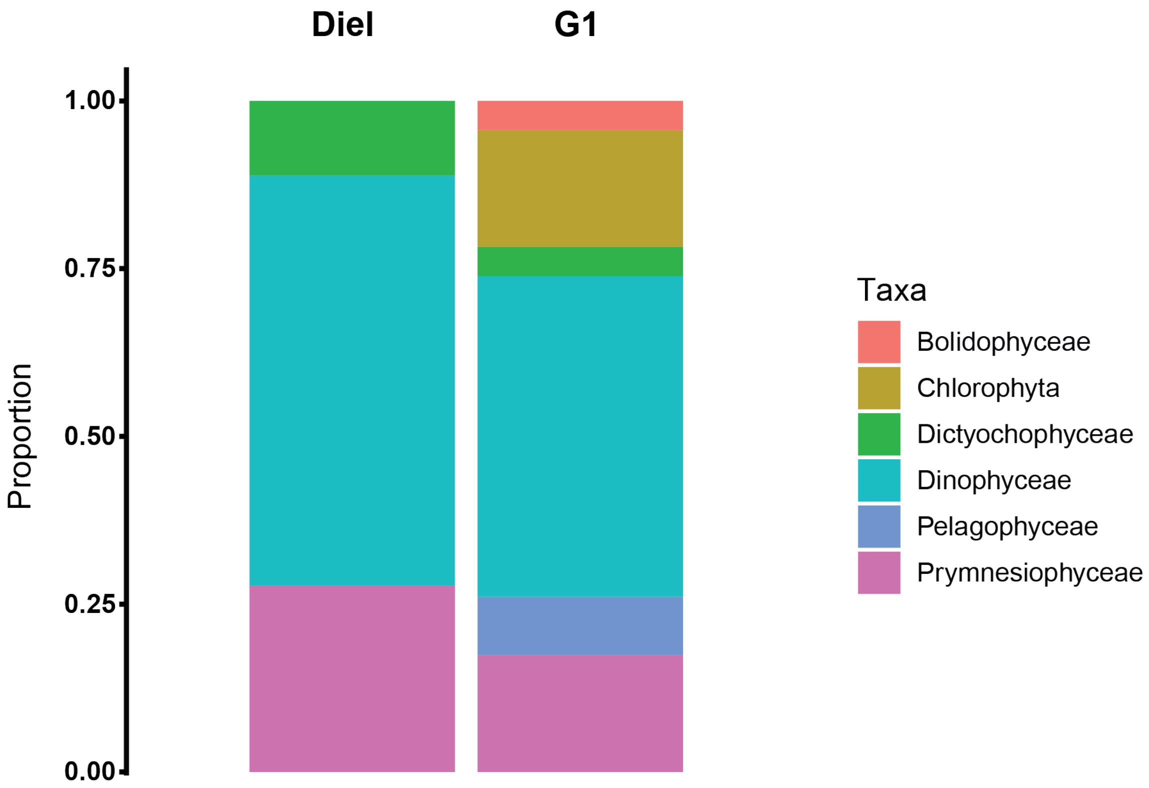

Figure S11. Taxonomic distribution of population transcriptome bins recovered from field data. In the Diel dataset, recovered taxonomic bins (cut-off 800 transcribed genes) were dominated by dinoflagellates and prymnesiophytes. Traxonomic bins from G1 cover more taxonomic diversity, but are still largely dominated by dinoflagellates and prymnesiophytes.

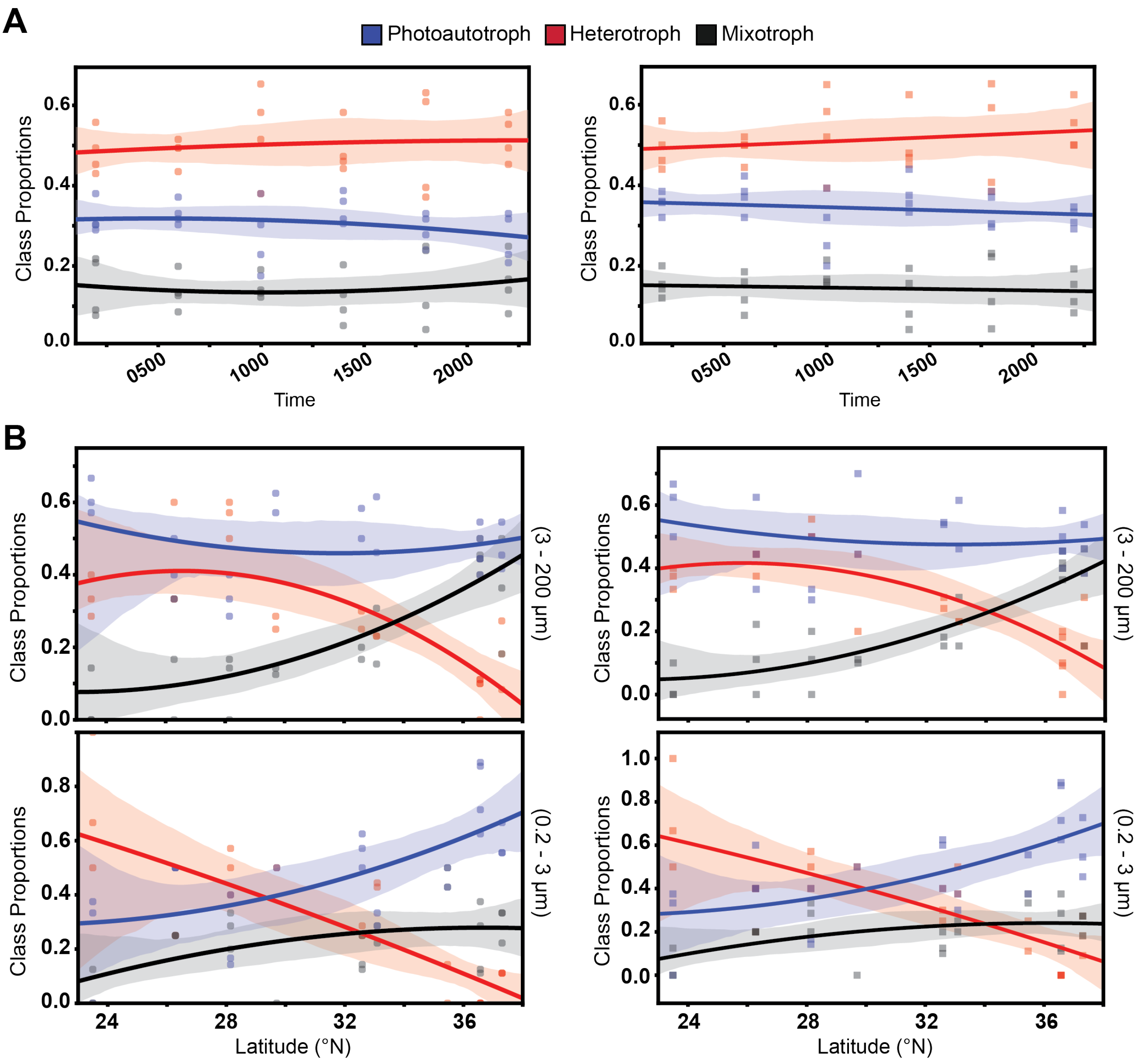

Figure S12. Comparison between aggregate trends in trophic mode prediction proportions for data when taxonomic bins with split phototrophy and heterotrophy were either removed (left) or retained (right) prior to analysis. (A) Trophic mode prediction proportions over the diel period are slightly different when applying our filtering criteria (SI Methods), but qualitative trends are the same. (B) Prediction proportions over the Gradients 1 transect in the small and large size classes are nearly identical with and without the removal of taxonomic bins with incongruous predictions.

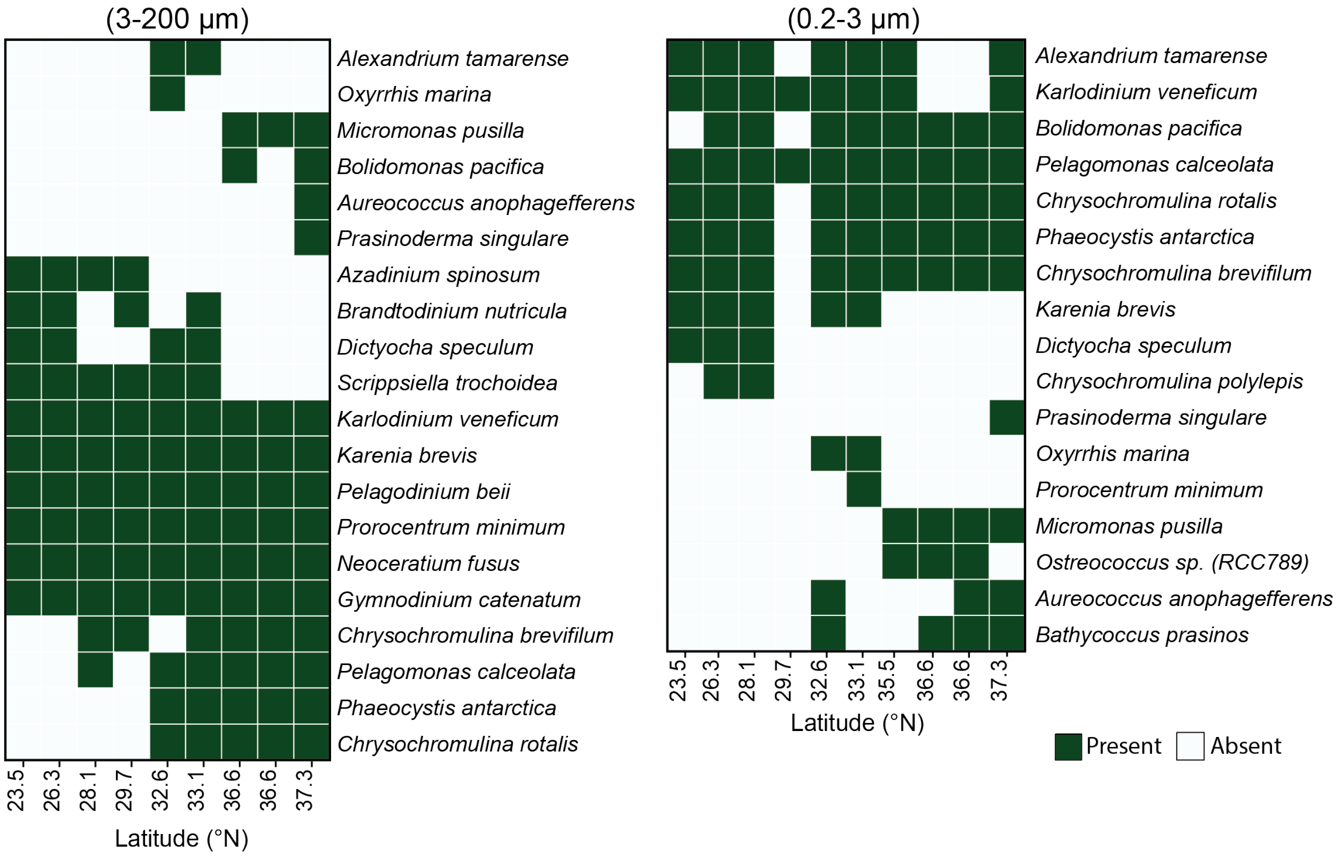

Figure S13. Presence/Absence of transcriptome bins meeting completeness criteria across the Gradients 1 transect. In both the small (0.2 – 3µm) and large (3 – 200µm) size fractions a core group of transcriptome bins were present over the entire Gradients 1 transect. Upon entry into the transition zone (near 30 ºN), new bins appear that are largely mixotrophic organisms (large size class) or a mixture of mixotrophic and phototrophic organisms (small size class).

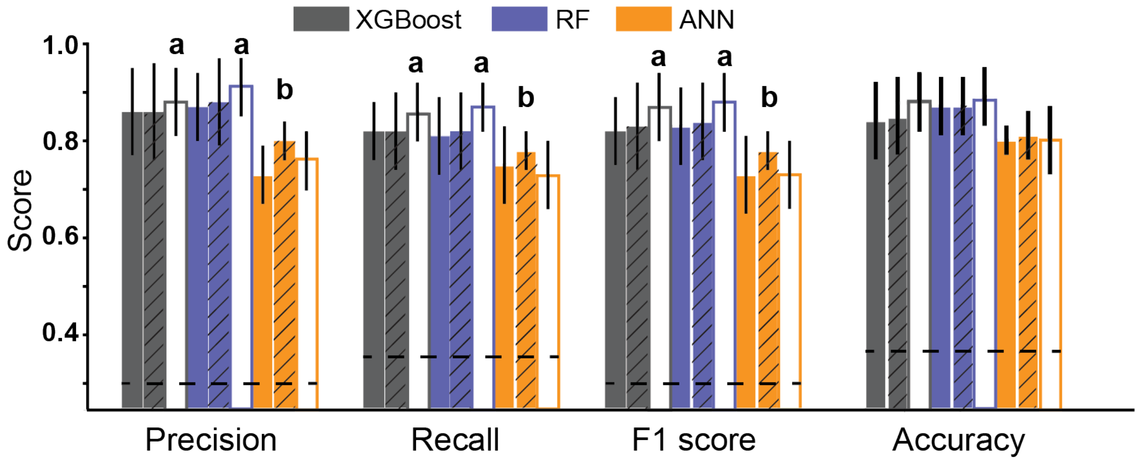

Figure S14. Precision, recall, F1 score, and Accuracy for the three evaluated models. The performance of Random Forest (RF), XGBoost, and an Artificial Neural Network (ANN), using all gene families present in the MMETSP (solid bars), the union of selected features ($f = 1046$; hatched bars), and the common set of selected features ($f = 120$; outlined bars). Significant differences in performance are indicated by lettering. (Kruskal-Wallis H-test, post-hoc pairwise Wilcoxon rank sum test; $p < 0.05$). Error bars indicate standard deviation. Dashed lines indicate performance of a dummy classifier on the same dataset. Measures were computed via 10-fold stratified cross validation.

Supplemental Files

File S1: Interactive t-SNE plot illustrating relationships between phylogeny and trophic mode in the t-SNE latent space. Individual transcriptomes are colored by trophic mode and shaped according to taxonomic group. The interactive tool allows users to hover over each data instance for more information about that transcriptome.

Supplemental Information: Appendix

Supplemental Note 1: Investigating factors that may impact prediction quality for environmental transcriptome bins.

We investigated several key data set characteristics that we hypothesized could impact classification of transcriptome bins derived from metatranscriptomes. These factors included:

1) The effect of transcriptional profile completeness on prediction outcome.
2) The effect of stochastic noise on prediction stability.
3) The potential for aggregation of closely-related species into a single taxonomic bin.
4) Increased sparsity in low abundance transcripts in metatranscriptome-derived taxonomic bins.

1. The effect of transcriptional profile completeness on prediction outcome

Estimating transcriptional profile completeness is challenging compared to genome completeness. Different gene suites may be transcribed depending on external abiotic and biotic conditions, as well as physiological processes. We examined the distribution of genes transcribed in the MMESTP by determining the number of gene families with non-zero transcript abundance in each transcriptome. A majority of transcriptomes had at least 800 gene families with non-zero transcript abundances (Fig. N1).

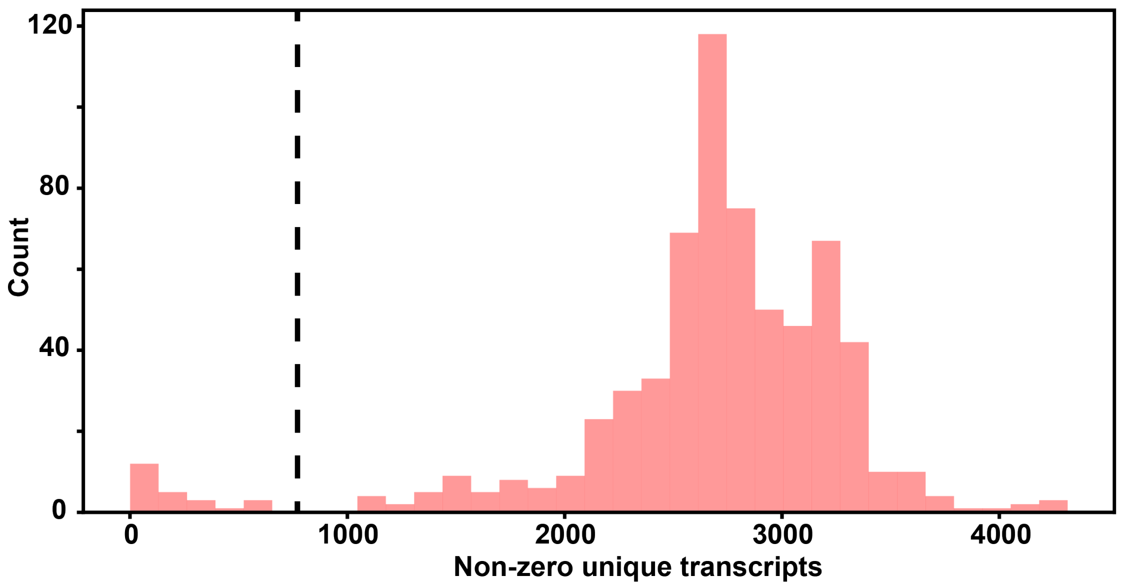

Figure N1. Histogram of unique transcripts with non-zero abundances in MMETSP transcriptomes. A cut-off for taxonomic bins was established at 800 non-zero elements, as the majority of MMETSP transcriptomes fall above this limit.

We then asked, how many transcribed genes were common between organisms in the MMETSP. We identified 306 'core' gene families that were transcribed in at least 95% of transcriptomes. One hundred thirty-five (44%) of these core genes were present within the union of features (*f* = 1046) selected via Mean Decrease in Accuracy. When transcriptional profiles were aggregated by taxonomic bin, all core gene families were transcribed within each taxonomic bin retrieved from station ALOHA.

We hypothesized that the probability of making a prediction for a given transcriptome within a replicate set that agreed with the majority of predictions for all transcriptome replicates would be related to transcriptome completeness as defined by the proportion of detected core gene families. After application of our filtering criteria (> 800 non-zero transcripts), the majority of transcriptomes were above 50% complete (Fig. N2A), with an average completeness of 72%, based on our core gene family analysis. The proportion of core genes detected was linearly related to the total number of detected genes (Fig. N2B). There was no relationship between model output and completeness in the Diel dataset, leading us to conclude that under the preprocessing and filtering scheme presented in this study, bin completeness, as measured by the presence of core gene families, did not affect model performance.

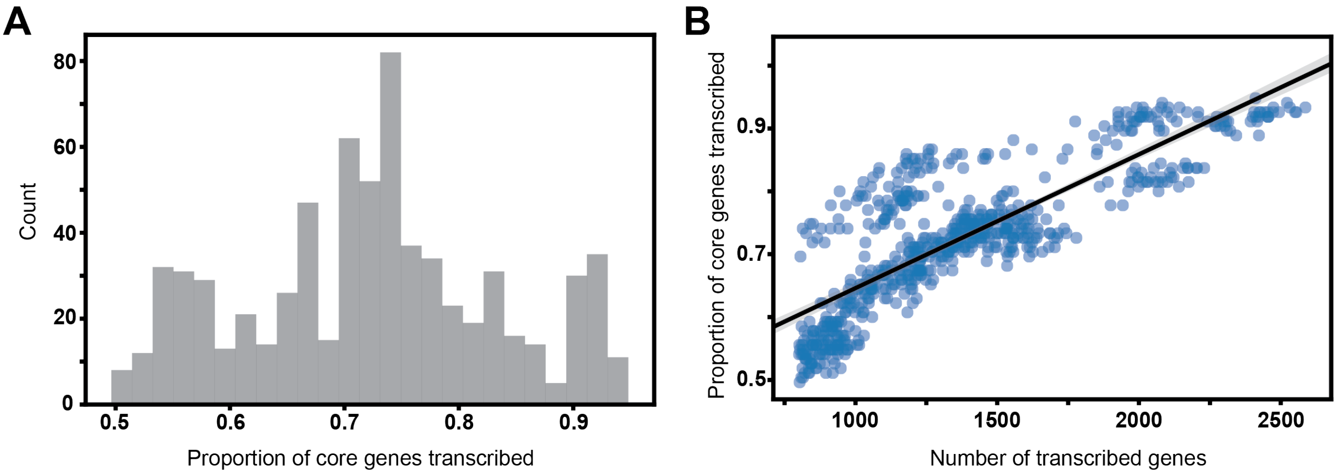

Figure N2. Estimates of transcriptome completeness for environmental species bins retrieved from Station ALOHA. (A) The presence of core genes in each transcriptome bin follows a normal distribution (μ=72%, σ=11%). (B) The proportion of core genes present in each environmental species bin is linearly related to the total number of transcribed genes in each transcriptome bin above the cut-off defined in this work.

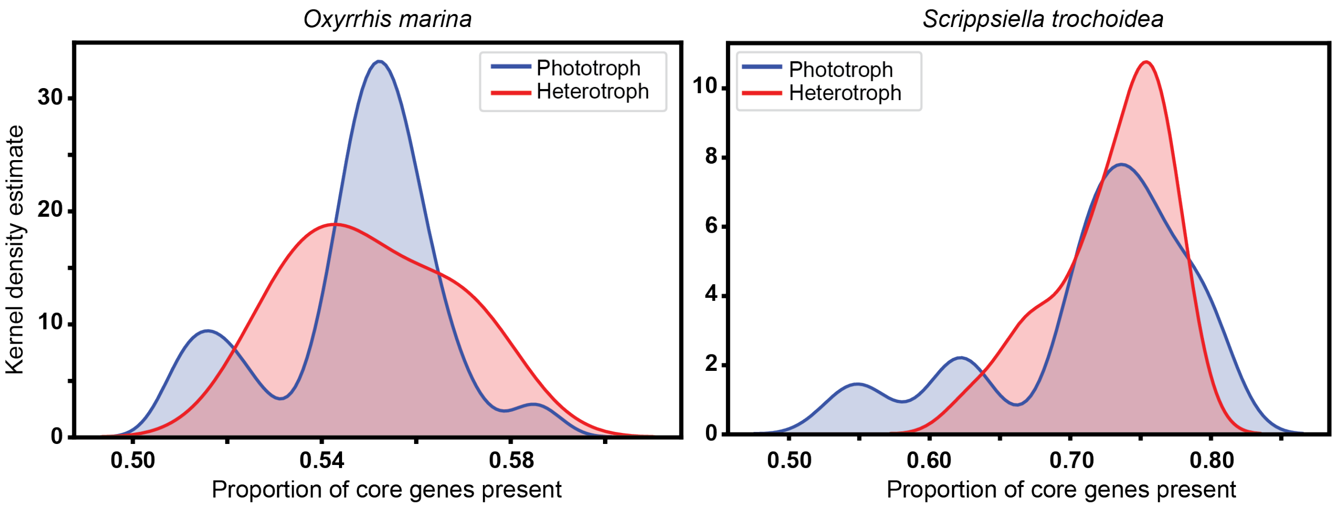

Figure N3. Transcriptional profile completeness does not affect model output for two species-level taxonomic bins with divergent predictions. Distributions of the proportion of core genes transcribed in transcriptomes that correspond to the same taxonomic bin with divergent predictions. Distributions overlap for bins with divergent phototrophy and heterotrophy predictions in the Dinoflagellates *Oxyrrhis marina* (*n* = 38) and *Scrippsiella trochoidea* (*n* = 45), suggesting that information loss throughout the sampling and analysis process is not a driving factor in model output for metatranscriptome-derived transcriptional profiles.

We focused on transcriptome completeness for two environmental dinoflagellate bins (*Oxyrrhis marina* and *Scrippsiella trochoidea*) that received conflicting heterotroph and phototroph predictions in the Diel dataset. There was no detected relationship between the proportion of transcribed core gene families in a given transcriptome and the resulting model prediction (Fig. N3). Thus, bin completeness, as assessed by the presence of core gene families, did not have any bearing on model performance.

2. The effect of stochastic noise on model output

We next assessed whether measurement noise in transcript abundance calculations could impact classification of transcriptomes, perhaps due to an unstable relationship with model decision boundaries. To interrogate this effect, we carried out a Monte Carlo style simulation for transcriptional profiles retrieved from the Diel dataset. The simulations were carried out using NumPy and Pandas in Python 3.7.

Random noise was introduced into transcriptome bin transcriptional profiles by performing the following operations:

1) Transcriptional profiles (*e*) corresponding to a particular taxonomic identifier were extracted from the Diel dataset resulting in a matrix of transcript abundance values of shape *number_bins, number_features*.

2) The median transcript abundance ($\bar{e}$) of each gene family in the union of selected features (f =1046) was computed for retrieved transcriptome bins.
3) A noise matrix (n) of the same shape was generated by randomly sampling from a normal distribution with means equal to the median transcript abundance values computed above and standard deviation equal to the square root of those median transcript abundance values.
4) A 'sign' matrix (s) was generated by randomly sampling from a uniform distribution defined over the interval [-1, 1). The purpose of this matrix was to randomize whether the noise computed above was added to or subtracted from each value in the transcriptome bin input matrix.
5) Finally, noisy transcriptional profiles were generated:

$$e_{mc} = e + \beta * n * s$$

where, β is a scaling factor that enables tuning of the magnitude of noise added to profiles.

We carried out this process 100 times for transcriptional profiles corresponding to each taxonomic identifier present in the Diel dataset and enumerated the number of instances where model output differed from baseline. For values of $\beta < 10$, model predictions were stable (Fig. N4); a high level of noise ($\beta = 10$) is likely equivalent to a sequence error well beyond those associated with today's short read sequencers or to a reduced ability to properly assign reads to the different environmental bins. Class-specific instability for simulations occurred only under conditions of $\beta = 10$. Transcriptome bins with a baseline prediction of mixotrophy changed on average 74% of the time when subjected to heavy noise ($\beta = 10$), compared to 30% and 25% for heterotrophy and phototrophy, respectively. This result is consistent with the feature landscape depicted in Fig. 1A and the notion that mixotrophy is the middle ground of the trophic spectrum. Given the high magnitude of noise required to alter model output, it appeared unlikely that measurement noise was a significant factor impacting prediction quality.

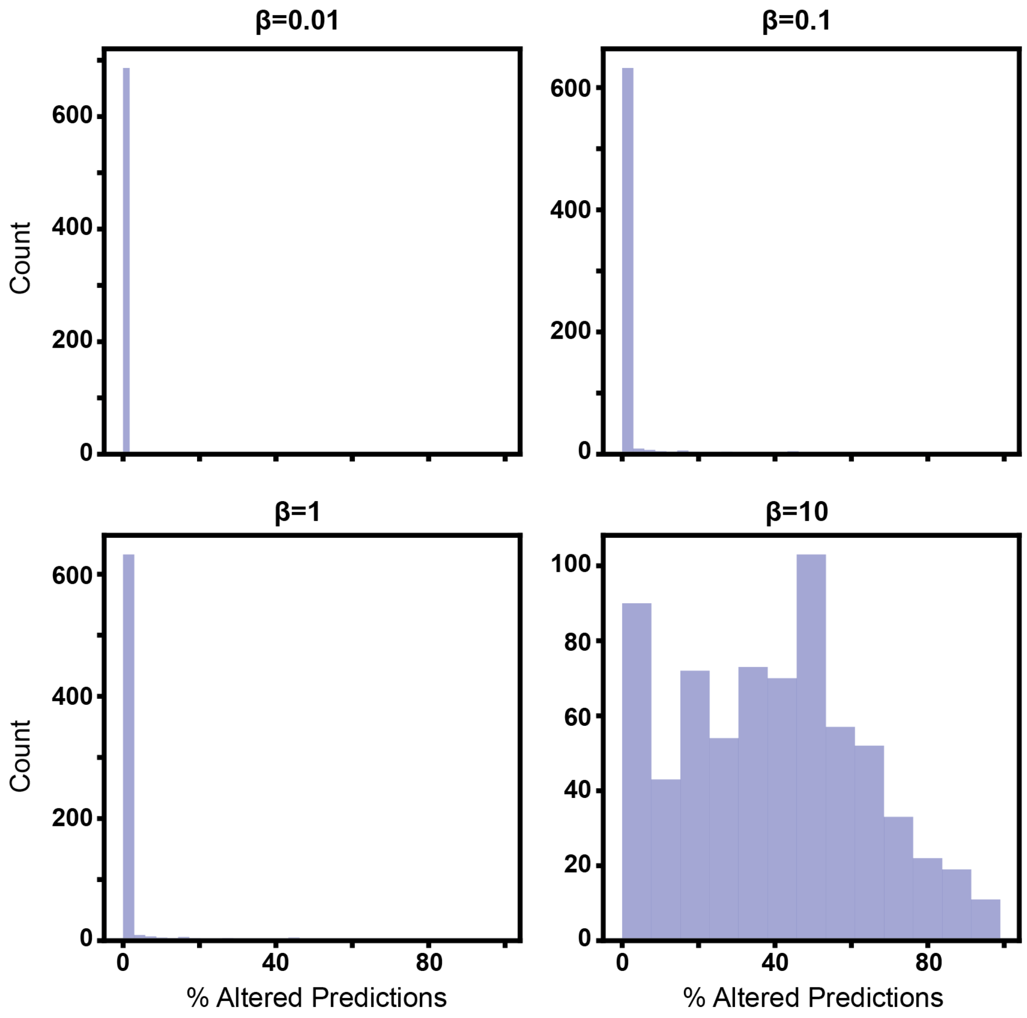

Figure N4. Monte Carlo sensitivity analysis shows that predictions only vary when input transcriptional profiles are subject to a large amount of noise. The scaling factor β directly impacts the quantity of noise injected into transcriptome bins prior to reclassification.

3. Investigating (sub-)species aggregation in metatranscriptome-derived taxonomic bins.

We evaluated whether multiple related species or sub-species were aggregated into taxonomic bins and if fluctuations in their abundance could drive differential bin classification. *Oxyrrhis marina* and *Scrippsiella troichoidea* transcriptome bins were classified as both heterotrophic and phototrophic. We hypothesized that evidence of species aggregation in transcriptome bins might be apparent in the distributions of e-values corresponding to taxonomic assignment. We constructed e-value distributions (Fig. N5) for *Scrippsiella trochoidea* and *Oxyrrhis marina* and compared those distributions to one resulting from *Alexandrium tamarense* (which received 100% consistent predictions). We observed no significant differences between the three distributions, suggesting that the lowest common ancestor algorithm was equally confident in its taxonomic assignments across the three bins and further suggesting that species aggregation was not a decisive factor in model performance.

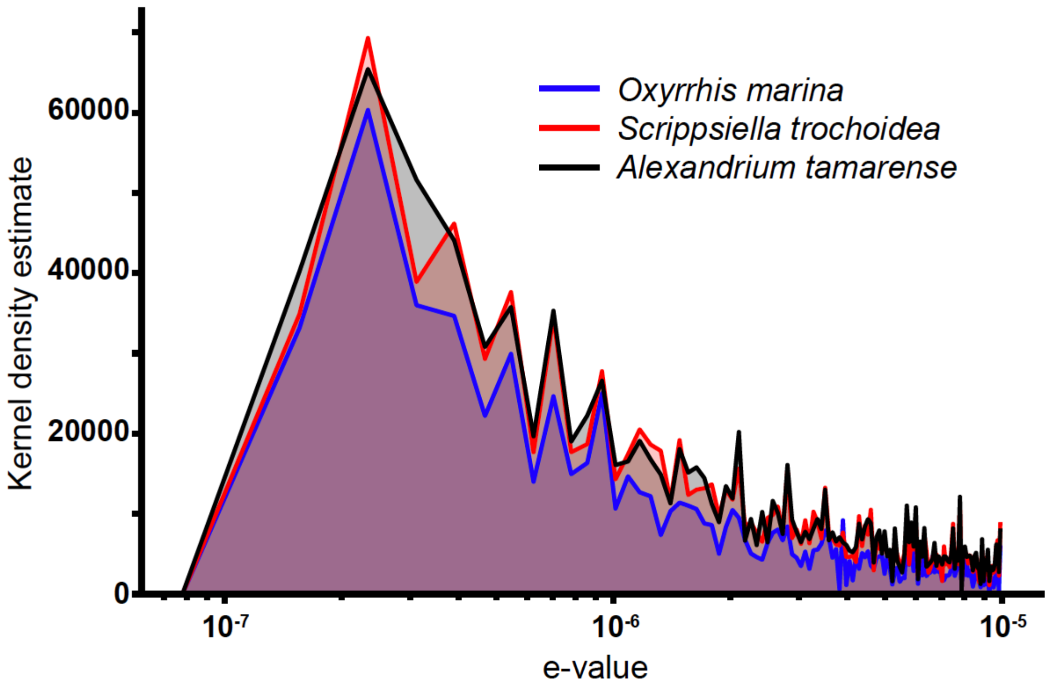

Figure N5. The e-value distributions for taxonomic placement of contigs belonging to three transcriptome bins. There are no apparent differences in the confidence of taxonomic placement between two bins with logically inconsistent predictions and one with complete prediction consistency.

In order to examine potential underlying reasons for these divergent predictions, we separated transcriptome bins from these environmental species by assigned class label and compared gene family transcript abundances in search of significantly differentially expressed families between predicted trophic modes. When comparing transcription of gene families between *Oxyrrhis marina* transcriptomes with divergent predictions, there were 7 significantly differentially abundant transcripts (Mann-Whitney U test, $p < 0.01$). For *Scrippsiella trochoidea* 19 significantly differentially abundant transcripts were detected (Mann-Whitney U test, $p < 0.01$). The majority of these genes were rare in our datasets and in most cases, were represented by a single contig across the entire Diel dataset. Contrary to our initial hypothesis, there was significantly less diversity in sequences belonging to these rare gene families when comparing contigs from the environment to those in the MMETSP. This led us to consider count sparsity as a potential driving factor in prediction quality.

4. Count sparsity in metatranscriptome-derived taxonomic bins

The environmental transcriptome bins carried forward in our study were on average 72% complete based on the presence of core gene families. We hypothesized that sparsity in non-core gene families might be the root cause of prediction divergence perhaps due to low transcript abundance for select features. We quantified sparsity by enumerating instances of zero transcript abundance on a per gene family basis for both the MMETSP data set and environmental transcriptome bins. As expected,

sparsity was more pronounced in transcriptional profiles derived from field data compared to the MMETSP (Fig. N6A). We therefore generated sparsity distributions for all taxonomic bins within the Diel dataset (Fig. N6B). Neither of the bins with divergent predictions (*Oxyrrhis marina* and *Scrippsiella trochoidea*) were obvious outliers in the sparsity distributions. Instead, two taxonomic bins highlighted through this analysis were *Dinophysis acuminata* and *Prymnesium parvum*, both of which consist of very few transcriptomes per taxonomic bin over the Diel experiment (*n=4*, *n=5* respectively). We evaluated whether removal of those gene families that tended to have low environmental transcript abundances from the training data would improve classification consistency with environmental transcriptomes. This process did not improve prediction consistency and in fact worsened it, suggesting that rare gene families are still of value when making predictions for environmental taxonomic bins.

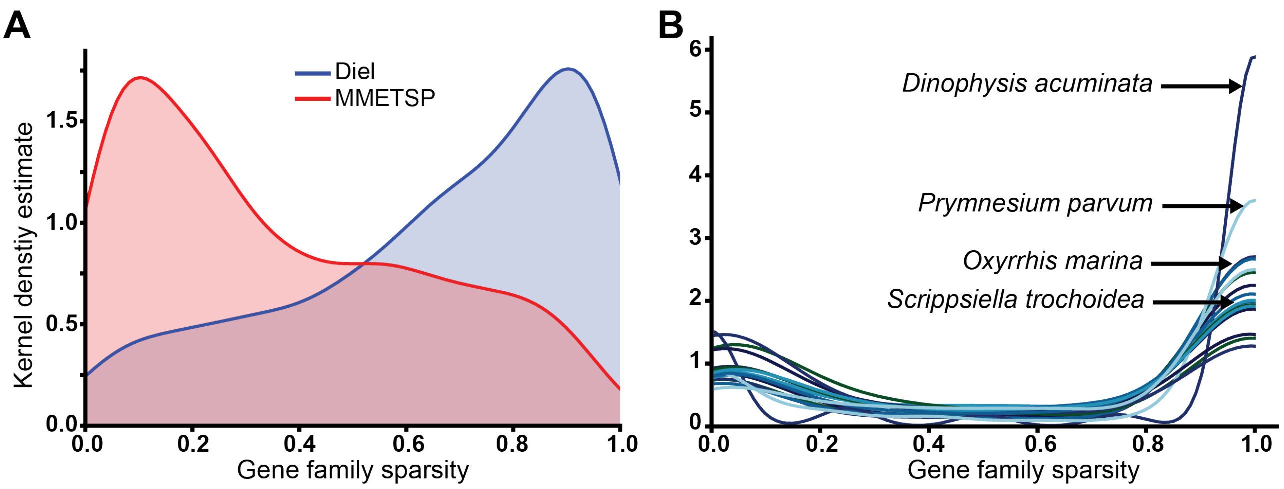

Figure N6. Count sparsity increases significantly in metatranscriptome-derived taxonomic bins. A sparsity value of 1 indicates no observed transcripts for a given gene family. (A) The Diel transcript count feature matrix contains a large number of zero values compared to the MMETSP. (B) Examining sparsity at the level of taxonomic species bins present in the Diel data set (different colored lines), the two organisms with the greatest transcriptome sparsity were *Dinophysis acuminata* and *Prymnesium parvum*, both of which were not abundant at the study site.

Supplemental Note 2: Calculation of respiration rates via metabolic theory.

The Metabolic Theory of Ecology predicts a temperature dependence for respiration in heterotrophic organisms and a combined dependence on temperature and light for photosynthetic organisms (16). Satellite sea surface temperature (14) and photosynthetically available radiation (15) were retrieved from Simons CMAP (SI Methods) and binned by latitude across Gradients 1. Sea surface temperature is assumed to be constant in the upper 15 m, consistent with CTD profiles at discrete stations. We assumed that the latitudinal gradient in the PAR extinction coefficient is negligible and hence use satellite-derived daily integrated surface PAR for all autotrophic respiration rate calculations. Using these data we applied an equation from (16) to calculate size-specific respiration rate in mmol O_2 d^{-1}. Respiration rate was calculated using the equation:

$$\ln(Respiration\ rate) = \ln(Nc) + \alpha \times \ln(M_i) - E \times \left({}^{1}/_{kT} \right) + \ln \left({}^{PAR}/_{k_m} + PAR \right)$$

Where N_c is a normalization constant, α is the allometric scaling exponent, M_i is the mass of a cell in Pg C, E is activation energy, k is Boltzmann's constant, and k_m is the Michaelis-Menten half-saturation constant. Values for k_m, α, and N_c were obtained via multiple regression in ref. (16). Absolute temperature is used and PAR is in mol photons m^{-2} d^{-1}. Both N_c and α differ between heterotrophs and photoautotrophs and the final term containing PAR is not included in heterotrophic respiration rate calculations. To estimate cell mass we used the scaling law $M \approx v^{0.712}$ (17) and a cell diameter of 3 µm. See (16) for more detailed discussion and Table 1 within for the above equation and parameter values.

Supplemental Note 3: Differential transcription analysis

Reads were mapped to reference transcriptomes using Salmon (2). The reference transcriptome collection consisted of two *Chrysochromulina* species isolated from off-shore of Hawaii (KB-HA01, AL-TEMP), four *Chrysochromulina* species from the MMETSP (*rotalis*, *polyepsis*, *brevifilum*, and *ercina*), *Prymnesium parvum*, and *Imantonia* sp. from the MMESTP. All selected reference prymnesiophyte transcriptomes were classified as mixotrophic by our model.

Differential transcription analysis was performed using the R package DESeq2 (18) with a significance cut-off of $p_{adj} < 0.05$. A total of 4 comparisons were made: subtropical gyre community ($n = 3$) vs subtropical gyre community + Nitrogen and Phosphorus (after 96 hr incubation; $n = 3$), subtropical gyre community bottle control (after 96 hr incubation; $n = 2$) vs subtropical gyre community + Nitrogen and Phosphorus (after 96 hr incubation; $n = 3$), North Pacific transition zone community control bottle (after 96 hrs; $n = 3$) vs North Pacific transition zone community + N,P,Fe (after 96 hr incubation; $n = 3$), and North Pacific transition zone community + Fe (after 96 hr incubation; $n = 3$) vs North Pacific transition zone community bottle control (after 96 hr incubation; $n = 3$). Across all

samples the number of reads that mapped to our prymnesiophyte references was relatively constant at ~ 300,000 – 400,000.

A total of 414 differentially transcribed genes (287 up, 127 down; Fig. N7A) were identified when the prymnesiophyte transcriptional profiles from the subtropical gyre were compared to the same community amended with 5 µM nitrate and 0.5 µM phosphate. The treatment and control were distinct along the first two principal components (Fig. N7B). For a summary of the functional categories that were differentially transcribed, see Fig. 5 of the main text. To evaluate potential bottle effects, we compared the bottle control (96 hr incubation) transcriptomes to the + Nitrogen and Phosphorus amendment transcriptomes. Fewer differentially transcribed genes (124 total; 60 up, 64 down; Fig. N8A) were detected, with samples still well-separated along the first two principal components (Fig. N8B). This is likely due to fewer samples in the control, which reduces the statistical power to identify significant differences between groups. When grouped into functional clusters (Dataset S18), the differentially expressed gene families followed similar patterns to those observed in the comparison between the subtropical gyre community ($t = 0$) and the +NP amendment ($t = 96$ hr) (Fig. N9; Main text Fig. 5).

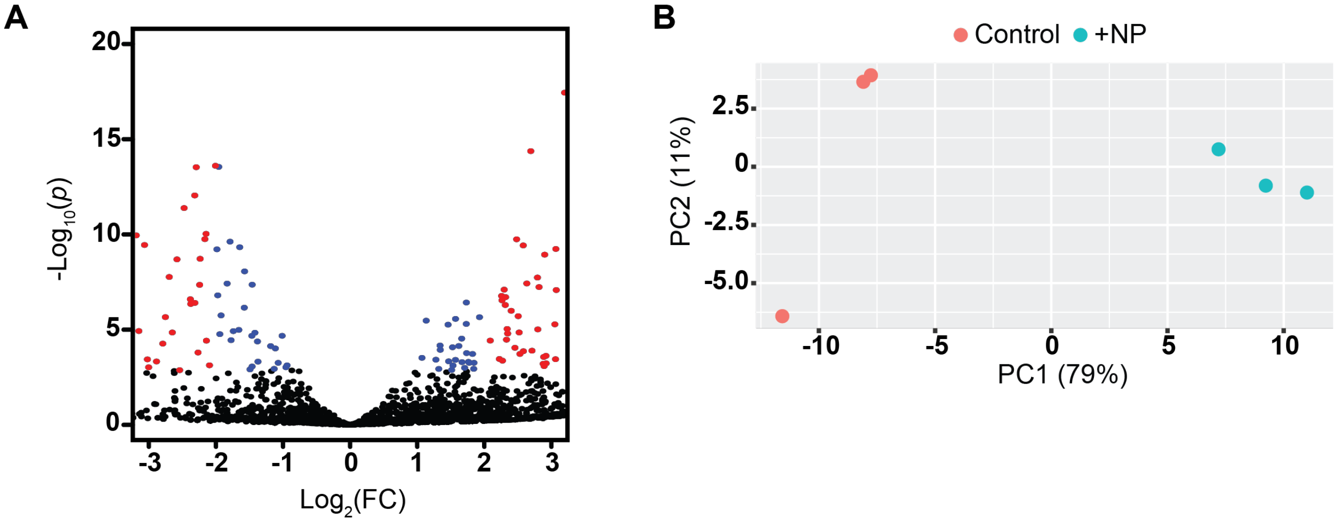

Figure N7. Differential transcription of genes upon amendment of subtropical gyre prymnesiophyte mixotrophs with nitrogen and phosphorus after 96 hr. (A) Volcano plot displaying the distribution of significantly differentially transcribed gene families. Blue points represent an adjusted p-value < 0.01 and red represents adjusted p-value < 0.01 and $|\mathrm{Log}_2(\mathrm{FC})| > 2.0$. (B) Treatment and control samples cluster distinctly along the first two principal components.

The differential transcription analysis of the North Pacific Transition Zone samples did not identify many significantly differentially transcribed genes. Comparing the +Fe treatment to the bottle control at 96 hrs, we observed only 2 genes (Dataset S15) that were differentially transcribed. The +NPFe treatment comparison yielded 14 differentially transcribed genes (Dataset S15).

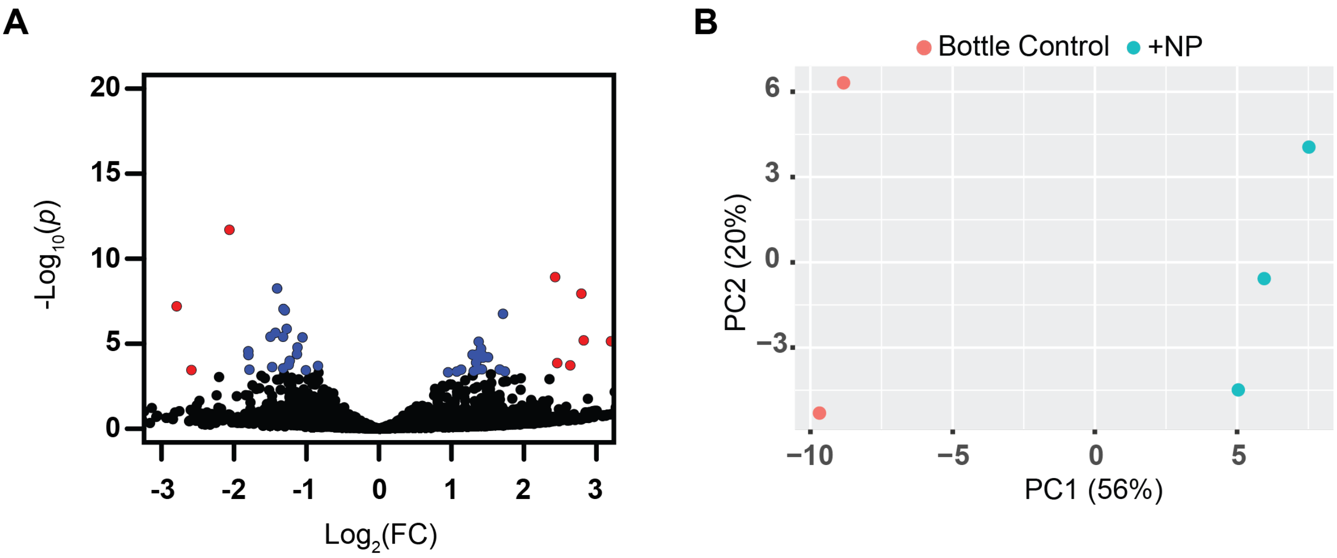

Figure N8. Differential transcription of genes by subtropical gyre mixotrophic prymnesiophytes upon amendment with nitrogen and phosphorus compared to the bottle control after 96 hr. (A) Volcano plot displaying the distribution of significantly differentially transcribed gene families. Blue points represent an adjusted p-value < 0.01 and red represents adjusted p-value < 0.01 and $|Log_2(FC)| > 2.0$. (B) Treatment and control samples cluster distinctly along the first two principal components.

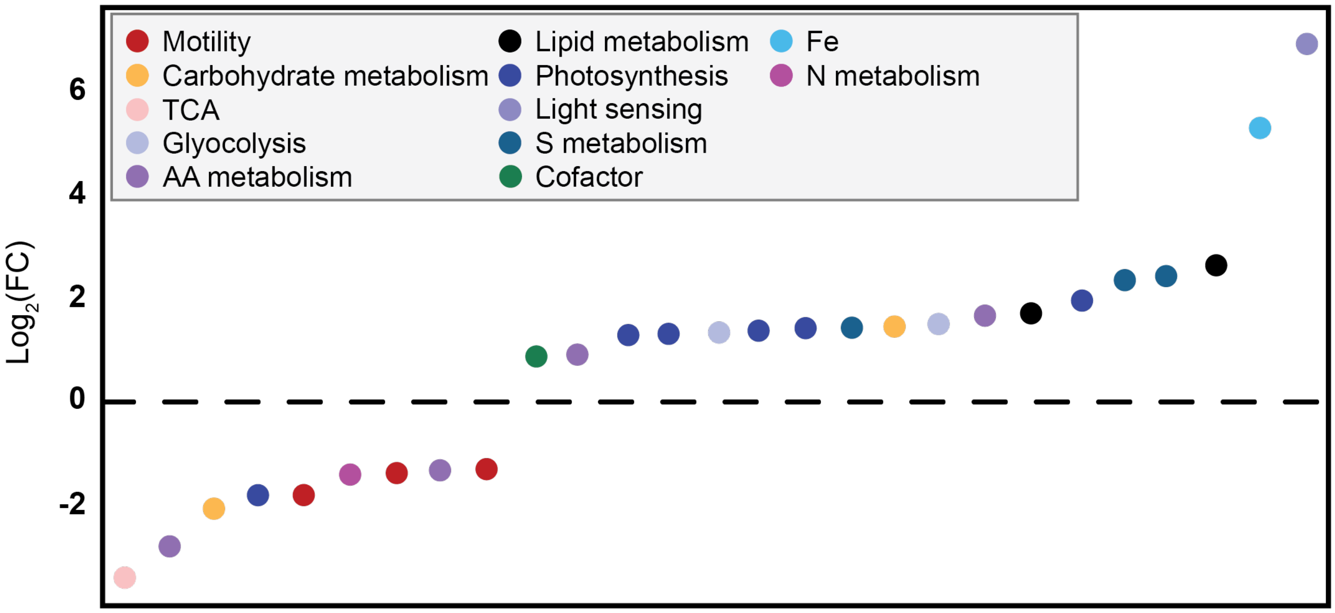

Figure N9. Differentially abundant transcripts in +NP treatment vs bottle control after 96 hrs. We observed similar transcriptional patterns to those present in the comparison with *in situ* subtropical gyre communities (*t=0*) when comparing the +NP bottle incubation prymnesiophyte expression profile to its associated bottle control (*t=96 hrs*).

Supplementary References

1. Johnson LK, Alexander H, & Brown CT (2018) Re-assembly, quality evaluation, and annotation of 678 microbial eukaryotic reference transcriptomes. *GigaScience* 8(4).
2. Patro R, Duggal G, Love MI, Irizarry RA, & Kingsford C (2017) Salmon provides fast and bias-aware quantification of transcript expression. *Nature Methods* 14(4):417-419.
3. Finn RD*, et al.* (2014) Pfam: the protein families database. *Nucleic acids research* 42(Database issue):D222-D230.
4. Dreiseitl S & Ohno-Machado L (2002) Logistic regression and artificial neural network classification models: a methodology review. *Journal of Biomedical Informatics* 35(5):352-359.
5. Ghojogh BC, Mark (2019) The Theory Behind Overfitting, Cross Validation, Regularization, Bagging, and Boosting: Tutorial. *arXiv*.
6. Katoh K, Misawa K, Kuma Ki, & Miyata T (2002) MAFFT: a novel method for rapid multiple sequence alignment based on fast Fourier transform. *Nucleic Acids Research* 30(14):3059-3066.
7. Stamatakis A (2014) RAxML version 8: a tool for phylogenetic analysis and post-analysis of large phylogenies. *Bioinformatics (Oxford, England)* 30(9):1312-1313.
8. Letunic I & Bork P (2019) Interactive Tree Of Life (iTOL) v4: recent updates and new developments. *Nucleic Acids Research* 47(W1):W256-W259.
9. Fore AG, Yueh SH, Tang W, Stiles BW, & Hayashi AK (2016) Combined Active/Passive Retrievals of Ocean Vector Wind and Sea Surface Salinity With SMAP. *IEEE Transactions on Geoscience and Remote Sensing* 54(12):7396-7404.
10. Aumont O, Éthé C, Tagliabue A, Bopp L, & Gehlen M (2015) PISCES-v2: An ocean biogeochemical model for carbon and ecosystem studies. *Geoscientific Model Development Discussions* 8(2).
11. Strickland JDH & Parsons TR (1972) A practical handbook of seawater analysis.
12. Ribalet F*, et al.* (2019) SeaFlow data v1, high-resolution abundance, size and biomass of small phytoplankton in the North Pacific. *Scientific Data* 6(1):277.
13. Swalwell JE, Ribalet F, & Armbrust EV (2011) SeaFlow: A novel underway flow-cytometer for continuous observations of phytoplankton in the ocean. *Limnology and Oceanography: Methods* 9(10):466-477.
14. OurOcean JPL (2010) GHRSST Level 4 G1SST Global Foundation Sea Surface Temperature Analysis. (NASA PO.DAAC).
15. NASA Goddard Space Flight Center OEL, Ocean Biology Processing Group (2018) Moderate-resolution Imaging Spectroradiometer (MODIS) Aqua Photosynthetically Available Radiation Data.
16. López-Urrutia Á, San Martin E, Harris RP, & Irigoien X (2006) Scaling the metabolic balance of the oceans. *Proceedings of the National Academy of Sciences* 103(23):8739.
17. Strathmann RR (1967) Estimating the organic carbon content of phytoplankton from cell volume or plasma volume. *Limnology and Oceanography* 12(3):411-418.
18. Love MI, Huber W, & Anders S (2014) Moderated estimation of fold change and dispersion for RNA-seq data with DESeq2. *Genome Biology* 15(12):550.
